# Carcinogen metabolism and bladder cancer: role of gut microbiota in disease and prevention

**DOI:** 10.3389/fcimb.2025.1727550

**Published:** 2026-01-30

**Authors:** Shen Pan, Hehe Zhu, Rui Yin, Jiaman Lin, Zhujun Wang, Wanlin Cui, Zhenhua Li, Bitian Liu

**Affiliations:** 1 Department of Nuclear Medicine, Shengjing Hospital of China Medical University, Shenyang, China; 2 Department of Urology, Shengjing Hospital of China Medical University, Shenyang, China; 3 Department of Microbiology and Immunology, Keio University School of Medicine, Tokyo, Japan; 4 Department of pediatrics, The first affiliated hospital of China Medical University, Shenyang, Liaoning, China

**Keywords:** bladder cancer, carcinogens, smoking, gut microbiome, metabolic regulation

## Abstract

Bladder cancer remains a significant global health concern, with environmental carcinogen exposure—particularly from tobacco-derived compounds such as aromatic amines, polycyclic aromatic hydrocarbons (PAHs), and nitrosamines—recognized as a primary etiological factor. These carcinogens undergo complex metabolic activation in the liver, bladder epithelium, and gut microbiota, generating reactive intermediates that initiate DNA damage, oxidative stress, and pro-tumorigenic signaling. This review synthesizes emerging evidence on how carcinogen-induced metabolic reprogramming contributes to bladder cancer initiation and progression, emphasizing the roles of key genetic pathways and metabolic enzymes involved in xenobiotic detoxification, DNA repair, and redox regulation. In parallel, we examine the influence of gut microbiota on carcinogen bioactivation and biotransformation, highlighting its dual role as both a metabolic modulator and a potential preventive target. We critically evaluate human observational data linking microbiome dysbiosis to bladder cancer risk, while addressing limitations such as small cohort sizes and confounders like diet and age. Finally, we discuss promising strategies for risk mitigation, including microbiome-directed interventions, dietary modulation, and chemopreventive agents that counteract carcinogenic effects. By integrating molecular oncology, toxicogenomics, and host-microbiome interactions, this review provides a mechanistic framework for understanding bladder cancer etiology and identifies novel opportunities for preventive and precision interventions.

## 1 Introduction

Bladder cancer is a malignancy with a high global incidence, particularly in industrialized countries, which imposes a significant economic burden on healthcare systems and society. Between 1990 and 2019, the global incidence of bladder cancer increased by 4%, with the U.S. reporting a 0.16% annual increase in transitional cell carcinoma (TCC) from 1973 to 2014, particularly during the early years of the study (1973–1987) (Al-Husseini et al., 2019; Safiri et al., 2021)​. Moreover, the diagnostic and therapeutic process often involves repeated surgeries, radiotherapy, and chemotherapy, which profoundly affects patients’ quality of life. Smoking and occupational exposure, as primary sources of carcinogens, account for approximately 60% of bladder cancer cases (Burger et al., 2013). Elevated recurrence rates and the necessity for long-term follow-up significantly contribute to the high costs associated with bladder cancer treatment, placing substantial strain on medical resources and increasing the financial burden on patients (Leal et al., 2016; Joyce et al., 2023; Clark et al., 2024). Targeting carcinogens either through source control or specific therapies is crucial for reducing the incidence of bladder cancer, thereby lowering healthcare costs and promoting long-term public health benefits (Mossanen and Gore, 2014; Fletcher et al., 2020; Lee et al., 2020; Sloan et al., 2020; Wéber et al., 2024).

Among the primary risk factors for bladder cancer, smoking, occupational exposure, and environmental pollution stand out as particularly significant. Smoking, including both traditional cigarettes and electronic cigarettes (e-cigarettes), stands as the most significant risk factor for bladder cancer, responsible for approximately 50% of cases (Kiriluk et al., 2012; Burger et al., 2013). Carcinogens such as aromatic amines, polycyclic aromatic hydrocarbons (PAHs), and nitrosamines released during tobacco combustion enter the body through inhalation, are metabolized, and subsequently excreted in urine. This process results in prolonged exposure of the bladder’s epithelial cells to these harmful substances, leading to DNA damage and initiating carcinogenesis (Leon et al., 2015; Sciannameo et al., 2019; Goyal et al., 2021). Recent studies have further confirmed the pivotal role of aromatic amines and other tobacco constituents in the development of bladder cancer (Alouini, 2024). Additionally, nitrosamine compounds, including N-nitrosodimethylamine (NDMA) and N-nitrosodiethylamine (NDEA), are recognized as critical pathogenic factors due to their potent carcinogenic effects (Kakizoe et al., 1979).

Occupational exposure to carcinogens in industries such as chemicals, dyes, rubber manufacturing, and petroleum processing is the second leading risk factor for urothelial bladder cancer (UBC) after smoking, accounting for approximately 20% of cases, primarily in industrial regions. Increased awareness and safety measures have significantly reduced exposure in recent years, but a study by Rushton et al. estimates that 7.1% of UBC cases in men are still attributed to occupational exposure (Boffetta et al., 1997; Rushton et al., 2010; Reed et al., 2020; Collatuzzo et al., 2024). Carcinogenic aromatic amines, such as aniline and β-naphthylamine, are extensively utilized in dye and rubber production, and numerous epidemiological studies have confirmed their association with an increased risk of bladder cancer among exposed workers (Cumberbatch et al., 2015; Cumberbatch et al., 2017). Similarly, chlorinated hydrocarbons like perchloroethylene (PCE) and trichloroethylene (TCE), commonly used in dry cleaning and industrial cleaning, are linked to a higher incidence of bladder cancer with long-term exposure (Wynder and Goldsmith, 1977; Abbaoui et al., 2018). Furthermore, inorganic arsenic (As^3+^ and As^5+^), which can be found at elevated levels in drinking water in certain regions, has been shown to correlate with increased bladder cancer risk due to chronic consumption (Wong et al., 2024). Notably, there is a synergistic effect between smoking and occupational exposure to these carcinogens, further heightening the risk of bladder cancer (Zhang et al., 1994).

Despite this understanding, current research has largely focused on bladder cancer treatment, while efforts to control carcinogen exposure at the source remain inadequate. Preventive strategies are more cost-effective than therapeutic interventions, as reducing carcinogen exposure or employing targeted detoxification therapies in high-risk populations can significantly lower bladder cancer incidence. Therefore, a systematic investigation of the primary carcinogens linked to bladder cancer, their mechanisms, metabolic pathways, and their impact on the tumor microenvironment (TME) is crucial for developing effective public health interventions. This review will analyze the mechanisms of bladder cancer carcinogens, identify key genes involved, explore the role of the gut microbiome, and summarize potential detoxifying foods or drugs, offering valuable insights for designing evidence-based prevention and control strategies.

## 2 Bladder cancer carcinogens

**Figure 1:**
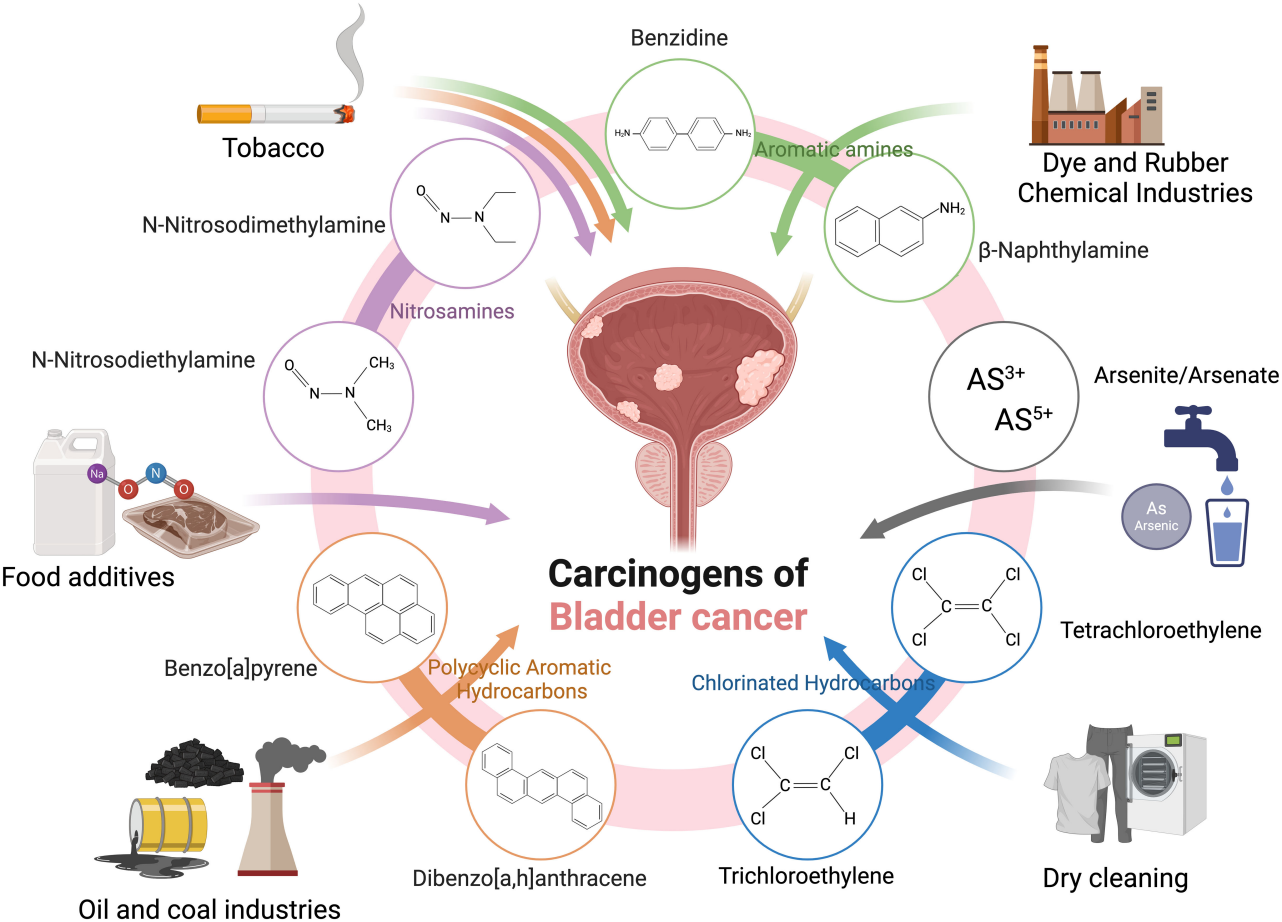
Key carcinogens associated with bladder cancer include nitrosamines, aromatic amines, polycyclic aromatic hydrocarbons, arsenic compounds, and chlorinated hydrocarbons, originating from sources such as tobacco, food additives, dry-cleaning solvents, industrial chemicals, and contaminated water.

Bladder cancer is linked to a wide range of carcinogens, including aromatic amines, PAHs, nitrosamines, arsenic, and organic chlorinated compounds, which promote carcinogenesis through DNA damage, oxidative stress, and inhibition of DNA repair. Traditionally associated with industrial exposures and tobacco products, newer sources of carcinogens are emerging (**Table 1**). Electronic cigarettes (e-cigarettes), like traditional smoking, expose users to bladder-specific carcinogens such as nitrosamines and volatile organic compounds, highlighting their potential cancer risk (**Table 2**). Additionally, microplastics, pervasive in water, air, and food, can carry toxicants like PAHs and heavy metals, which may accumulate in the urinary system. Per- and polyfluoroalkyl substances (PFASs), persistent in drinking water and industrial products, and chlorinated disinfection by-products are also gaining recognition as risk factors. These developments underscore the need for updated strategies to address both traditional and modern exposures contributing to bladder cancer (**Figure 1**).

**Table 1:** Evolution of airborne carcinogens across eras.

Era	Key Sources of air pollution	Primary carcinogens	Characteristics
Pre-Industrial	Biomass burning Cooking smoke	PAHs Soot	Indoor air pollution dominated, with localized impact.
Industrial Revolution	Coal combustion Smelting industries	PAHs Benzene Arsenic	Outdoor pollution from coal combustion intensified.
Early 20th Century	Coal-fired power plants Residential heating	Benzene Formaldehyde Lead	Urban smog events began to highlight health risks.
Mid-20th Century	Cars (leaded gasoline) Industrial processes	Benzene NO_X_ Heavy metals (e.g., lead)	Photochemical smog and industrial expansion worsened.
Late 20th Century	Diesel vehicles Coal power plants	PM2.5 Diesel exhaust Formaldehyde	Regulatory action reduced emissions in developed nations, but urban pollution persisted globally.
Late 20th Century	Diesel vehicles Coal power plants	PM2.5 Diesel exhaust Formaldehyde	Regulatory action reduced emissions in developed nations, but urban pollution persisted globally.
21st Century	Motor vehicles Wildfires Industries	PM2.5/PM10 Volatile Organic Compounds Heavy metals	Increased global emissions due to industrialization and climate-related wildfires.

**Table 2:** E-cigarettes vs. traditional cigarettes: key differences.

Aspect	E-cigarettes	Traditional cigarettes	Key comparison
Carcinogenic Compounds	Nicotine-derived nitrosamines (NNK, NNAL); Volatile organic compounds (formaldehyde, acrolein); Metals (cadmium, nickel)	Combustion by-products (benzo[a]pyrene, benzene, PAHs); Tobacco-specific nitrosamines (NNK, NNAL)	E-cigarettes reduce combustion-related toxins but still contain harmful and carcinogenic chemicals.
Delivery Mechanism	Vaporization at 200–300°C	Combustion at 600–900°C	Vaporization avoids combustion toxins but produces harmful thermal degradation by-products.
Health Risks	DNA damage; Oxidative stress- Bladder and lung effects (e.g., hyperplasia)	Lung, bladder, and oral cancers; Chronic diseases (e.g., COPD)	Both contribute to oxidative stress and DNA damage; long-term e-cigarette risks remain unclear.
Addictive Substances	Nicotine with added flavorings	Nicotine with tobacco alkaloids	Both are highly addictive; flavored e-liquids may appeal more to youth.
Regulation	Varies widely; marketed as safer alternatives	Strict regulation globally	E-cigarettes face fewer regulations despite growing evidence of health risks.

NNK: Nicotine-derived nitrosamine ketone.

NNAL: Nicotine-derived nitrosamine 1’-oxide.

COPD: Chronic Obstructive Pulmonary Disease.

### 2.1 Aromatic amines

Aromatic amines have garnered significant attention as bladder carcinogens in occupational disease and public health research. Key compounds in this category include aniline, β-naphthylamine, 4-aminobiphenyl, and 3,3’-dichlorobenzidine, which are widely used in industries such as dye, rubber, plastic, and pesticide production. Exposure to these compounds, particularly through inhalation or prolonged contact with their metabolic byproducts, markedly increases the risk of developing bladder cancer (Cohen, 1983; Imaoka et al., 1997; Tao et al., 2013). Benzidine, one of the most extensively studied carcinogens, serves as a crucial intermediate in dye and plastic production. Once in the body, benzidine undergoes metabolic activation in the liver, forming DNA adducts in bladder epithelial cells and causing DNA damage. Strong correlations have been observed between benzidine exposure and bladder cancer incidence, especially among workers with long-term exposure, whose risk is several times higher than that of the general population (Golka et al., 2004a; Wang et al., 2019). Similarly, β-naphthylamine is a potent carcinogen primarily used in dye and rubber manufacturing. Its carcinogenic potential was recognized in the twentieth century, linking it closely to bladder cancer incidence (Kogevinas et al., 2003; Habil et al., 2022). 4-Aminobiphenyl, historically used in the dye, rubber, and pesticide industries, is also a recognized carcinogen. Workers with prolonged exposure to 4-aminobiphenyl exhibit significantly higher bladder cancer rates compared to the general population (Tao et al., 2013; Wang et al., 2019). 3,3’-Dichlorobenzidine is another aromatic amine associated with bladder cancer, primarily used in pigment and dye production, and has been shown to increase DNA damage risk in bladder epithelial cells (Ghosal and Iba, 1990; Imaoka et al., 1997; Golka et al., 2004a). Other aromatic amines, such as 2-methoxy-4-aminobiphenyl and m-phenylenediamine, are suspected of having carcinogenic properties related to bladder cancer, but research on these compounds is still limited (Golka et al., 2004a).

### 2.2 PAHs

PAHs are highly carcinogenic compounds consisting of multiple fused benzene rings. These compounds primarily enter the body through smoking, industrial pollution, and environmental exposure. Smoking is a significant risk factor for bladder cancer, as smokers are exposed to elevated levels of PAH metabolites, thereby increasing their risk of developing the disease (Trédaniel et al., 1993; Jin et al., 2017). Among the PAHs, benzo[a]pyrene is one of the most extensively studied carcinogens, commonly found in cigarette smoke, vehicle exhaust, and industrial emissions. After metabolic activation, benzo[a]pyrene produces reactive intermediates that form DNA adducts, leading to damage that can result in gene mutations and initiate cancer development (Moore et al., 1982; Hathway, 2000; Verma et al., 2017). Dibenzo[a,h]anthracene is another highly carcinogenic PAH; its metabolites also cause DNA damage and mutations in bladder epithelial cells (Mastrangelo et al., 1996). Additionally, pyrene and phenanthrene, which are prevalent in industrial emissions and tobacco smoke, have been shown to generate metabolites that induce DNA damage and promote carcinogenesis (Xu et al., 2006; Madrid et al., 2020; Choi et al., 2023). Other notable PAHs include anthracene and fluoranthene, which are released during the combustion of organic materials, such as from smoking and vehicle exhaust, and exhibit carcinogenic effects on bladder epithelial cells (Boström et al., 2002; Kim et al., 2013).

### 2.3 Nitrosamines

Nitrosamines are significant carcinogenic agents in bladder cancer, widely found in food, tobacco products, and industrial chemicals. Their potent carcinogenicity and widespread environmental exposure have raised considerable concern. Nitrosamines induce direct DNA damage through alkylation mechanisms, forming stable DNA adducts that can lead to mutations and facilitate malignant transformation. NDMA and NDEA are among the most extensively studied nitrosamines, entering the body primarily through food processing and tobacco products. NDMA metabolism generates highly reactive methylating metabolites (e.g., O6-methylguanine, m6G), which bind to DNA, resulting in base mutations and an increased risk of bladder cancer (Anderson et al., 1996). Similarly, NDEA produces alkylating intermediates through comparable metabolic pathways, significantly elevating the incidence of bladder cancer, particularly at high exposure levels (Wiench et al., 1992; Cai et al., 2023). Other nitrosamines, such as N-nitrosopyrrolidine (NPYR) and N-nitrosomorpholine (NMOR), are commonly found in specific foods (e.g., cured meats), tobacco smoke, and industrial waste (Chen et al., 2019; Schrenk et al., 2023; Chen et al., 2024). In summary, nitrosamines play a crucial role in the carcinogenesis of bladder cancer. The diverse types of nitrosamines, their metabolic pathways, and their accumulation in the bladder position them as critical targets for research and prevention strategies related to this malignancy.

### 2.4 Arsenic and its compounds

Arsenic and its compounds are significant carcinogenic agents associated with bladder cancer, with exposure occurring through various routes, including drinking water, air pollution, food, and industrial waste (Christoforidou et al., 2013; Tsuji et al., 2014). The mechanisms of arsenic carcinogenicity are complex and include the induction of oxidative stress, disruption of DNA repair pathways, and alterations in DNA methylation. Arsenic trioxide, a common inorganic arsenic compound, inhibits the activity of key enzymes involved in DNA repair, leading to the accumulation of DNA damage and an increased risk of developing bladder cancer. Furthermore, exposure to arsenic trioxide induces oxidative stress responses in cells, which can further enhance carcinogenesis (Hour et al., 2004; Kurosawa et al., 2023). Arsenate can be reduced to trivalent arsenic within the body, resulting in heightened toxicity. This compound disrupts normal metabolic processes in bladder epithelial cells, inhibits DNA repair enzyme activity, and promotes the accumulation of base mismatches in DNA, ultimately resulting in mutations and cancer development (Muenyi et al., 2015; Tsai et al., 2021). Recent research has also revealed that arsenate can influence gene expression through epigenetic mechanisms, particularly affecting pathways associated with cancer, thereby increasing its carcinogenic potential (Marsit et al., 2006; Bustaffa et al., 2014; Yamamoto et al., 2023).

### 2.5 Organic chlorinated compounds and solvents

Organic chlorinated compounds constitute another significant class of bladder carcinogens, primarily entering the environment through industrial emissions, pesticide use, and plastic products. Notable examples include hexachlorobutadiene and polychlorinated biphenyls (PCBs). Hexachlorobutadiene, utilized in the rubber industry and pesticide manufacturing, produces metabolites that accumulate in bladder cells and induce gene mutations by binding to DNA, thus posing a potential risk for bladder cancer (Zhang et al., 2019; Lu et al., 2021; Yang et al., 2023; Zhao et al., 2024b). PCBs, widely used in electronic devices and construction materials, have metabolites that can inflict DNA damage through oxidative stress, leading to mutations and subsequent carcinogenesis in bladder epithelial cells (Beyer and Biziuk, 2009; Grimm et al., 2015; Zarerad et al., 2023). Additionally, organic solvents commonly employed in dry cleaning, such as PCE and TCE, are also significant contributors to bladder cancer risk (Weiss, 1995; Vlaanderen et al., 2014; Callahan et al., 2019). PCE typically enters the body through inhalation and dermal contact, where it is metabolized in the liver to form carcinogenic intermediates. These intermediates can accumulate in the bladder, resulting in DNA damage and increased risk of bladder cancer (Lash and Parker, 2001). TCE, a prevalent industrial solvent, similarly produces metabolites that bind to DNA, affecting cellular proliferation and differentiation, ultimately leading to cancer (Motohashi et al., 1999).

## 3 Carcinogenic mechanism

Environmental carcinogens contribute to the development of bladder cancer through multiple mechanisms, such as DNA adduct formation, epigenetic regulation alterations, and genetic susceptibility. The interaction between metabolic enzyme polymorphisms and environmental exposures creates significant variability in individual susceptibility, thereby increasing the risk of cancer. While these mechanisms are well-supported by *in vitro* and animal studies, human epidemiological data show variability due to confounders like exposure dose and genetic background, highlighting the need for larger, prospective cohorts to resolve conflicting findings.

### 3.1 Inducing gene mutations via DNA adduct formation

Many carcinogens linked to bladder cancer promote gene mutations through the formation of DNA adducts, leading to malignant cell transformation. Aromatic amines (e.g., aniline, β-naphthylamine) are metabolically activated in the liver via pathways involving N-acetyltransferases (NAT) and N-hydroxylation. The resulting N-hydroxy derivatives can form adducts with guanine in DNA, disrupting base pairing and compromising the structural integrity of DNA. This process results in mutations, particularly in critical tumor suppressor genes like TP53, significantly elevating the risk of bladder cancer (Vineis and Pirastu, 1997; Golka et al., 2004b; Neumann, 2010; Besaratinia and Tommasi, 2013). Similarly, PAHs (e.g., benzo[a]pyrene and dibenz[a,h]anthracene) are metabolized by cytochrome P450 (CYP450) enzymes (e.g., CYP1A1) into highly reactive benzo[a]pyrene diol epoxides (BPDE). These metabolites also bind to DNA, forming adducts that lead to double-strand breaks and gene mutations. The accumulation of such adducts in bladder epithelial cells represents a crucial initiating event in bladder carcinogenesis (Poirier, 1997; Roos and Bolt, 2005; McCarty et al., 2009; Jin et al., 2017; Barnes et al., 2018; Stading et al., 2021).

### 3.2 Promoting malignancy through epigenetic dysregulation

The incidence of bladder cancer is closely associated with epigenetic dysregulation induced by various environmental carcinogens (Han et al., 2012; Das and Ravi, 2022; Wang et al., 2023; Zhao et al., 2024a). The metabolic product of PAHs, particularly BPDE, not only forms covalent adducts with DNA, resulting in genomic instability, but also regulates the expression of oncogenes and tumor suppressor genes through epigenetic mechanisms (Li et al., 2007; Zhao et al., 2024a). Specifically, BPDE can induce global hypomethylation by inhibiting DNA methyltransferases (DNMTs), which leads to the upregulation of oncogene expression and downregulation of tumor suppressor gene function, thus promoting the proliferation and metastasis of bladder cancer cells (Bukowska and Sicińska, 2021; Zhao et al., 2024a; Zou et al., 2024). Furthermore, BPDE alters histone methylation and acetylation modifications, further changing chromatin structure and activating oncogenes (Sculley and Zytkovicz, 1983; Kurokawa and MacLeod, 1985; Mann et al., 1997; Wang et al., 2020). Additionally, nitrosamines (e.g., NDMA) generate alkylating intermediates that damage the genome through DNA adducts such as m6G, inducing abnormal DNA methylation and increasing the risk of malignant transformation in bladder cancer cells (Anderson et al., 1996; Okaru and Lachenmeier, 2021).

### 3.3 Oxidative stress

Oxidative stress is considered a significant contributor to the carcinogenic mechanisms involved in bladder cancer. Various environmental carcinogens, including arsenic and its compounds, PCBs, as well as the dry-cleaning solvents PCE and TCE, induce the generation of reactive oxygen species (ROS) within the body. This ROS production leads to oxidative damage, which promotes the development of bladder cancer. Arsenic exposure has been shown to significantly elevate intracellular ROS levels, and an excess of ROS can cause oxidative damage to DNA, resulting in modifications to DNA bases and double-strand breaks. Such damage activates intracellular stress responses that disrupt cell cycle regulation, contributing to genomic instability and carcinogenesis (Tchounwou et al., 2004; Ozturk et al., 2022; Das, 2024). As widespread persistent organic pollutants, PCBs also contribute to oxidative stress by increasing ROS levels in bladder epithelial cells, thereby damaging DNA structure and exacerbating the pathological progression of bladder cancer (Carpenter, 2006; Montano et al., 2022). However, conflicting evidence exists; some studies suggest ROS levels vary by exposure duration, and antioxidant interventions show inconsistent efficacy in human trials (Reuter et al., 2010; Prasad et al., 2017; Di Bona et al., 2024).

Furthermore, PCE and TCE, commonly used in the dry-cleaning industry, are metabolized into various oxidative intermediates. These intermediates are activated by the CYP450 enzyme system present in the liver and bladder epithelial cells, leading to substantial ROS production (Bruckner et al., 1989; Lash et al., 2000). The resulting ROS can oxidize DNA bases, proteins, and lipid components of cell membranes, leading to double-strand breaks and base damage. This oxidative damage triggers dysregulation of apoptotic pathways and further exacerbates genomic instability, significantly increasing the risk of bladder cancer (Lock and Reed, 2006; Guyton et al., 2014; Rusyn et al., 2014; Zhu et al., 2024). Research indicates that oxidative stress responses can promote the malignant progression of bladder cancer by inducing inflammation, inhibiting antioxidant defense systems, and activating oncogenes, though these effects may be modulated by individual genetic factors (Reuter et al., 2010; Prasad et al., 2017; Di Bona et al., 2024).

### 3.4 Genetic susceptibility and metabolic polymorphisms in carcinogenesis

The interaction between genetic and environmental factors is particularly pronounced in the carcinogenesis of bladder cancer. Numerous epidemiological studies have demonstrated that exposure to carcinogens poses a greater risk to genetically susceptible individuals. This interaction may affect key cellular processes such as DNA repair efficiency, cell proliferation control, and metabolic balance. Polymorphisms in certain metabolic genes can influence the metabolic efficiency of carcinogens, thereby increasing the risk of bladder cancer. For instance, NATs are closely associated with the metabolism of aromatic amines (Minchin and Butcher, 2015; Conway et al., 2020). The polymorphisms in NAT genes determine the rate at which individuals metabolize carcinogens; slow metabolizers are more vulnerable to the toxic effects of these substances, heightening the risk of bladder cancer (Badawi et al., 1995; Hein et al., 2000). The CYP450 enzyme CYP1A2 plays a crucial role in the primary metabolism of aromatic amine carcinogens, and its polymorphisms have been linked to genetic susceptibility to bladder cancer (Pavanello et al., 2010). High-activity variant alleles of CYP1A2 can enhance the activation of carcinogens, increasing the risk of bladder cancer in exposed individuals. Epidemiological studies have identified a significantly elevated risk of bladder cancer among smokers who carry the high-activity genotype of CYP1A2 (Pavanello et al., 2010). Glutathione S-transferase M1 (GSTM1) is another important metabolic enzyme gene that catalyzes the conjugation of glutathione with electrophilic compounds, converting activated carcinogens into inactive, water-soluble substances for excretion. However, deletion mutations of the GSTM1 gene are prevalent in a substantial portion of the population. Individuals lacking this enzyme cannot effectively eliminate carcinogens from their bodies, which increases the risk of bladder cancer (Zhao et al., 2007; Salinas-Sánchez et al., 2011). The absence of the GSTM1 gene is highly associated with the incidence of bladder cancer in smokers and individuals with occupational exposure to aromatic amines and other industrial chemicals (Hung et al., 2004; García-Closas et al., 2005; Yu et al., 2016). Critically, meta-analyses show inconsistent associations across populations, potentially due to ethnic differences and small sample sizes in some studies (Hung et al., 2004; García-Closas et al., 2005; Zhao et al., 2007; Salinas-Sánchez et al., 2011; Yu et al., 2016).

### 3.5 Inhibition of DNA repair

Bladder cancer is closely linked to mutations or polymorphisms in genes involved in DNA repair mechanisms. These repair processes are vital for maintaining genomic stability and preventing cellular transformation. Key genes in the nucleotide excision repair (NER) pathway, such as Excision Repair Cross-Complementing 2 (ERCC2) and Xeroderma Pigmentosum Group C (XPC), are responsible for identifying and repairing DNA damage induced by ultraviolet (UV) radiation and various chemical carcinogens. When mutations or functional polymorphisms occur in these genes, the efficiency of DNA damage repair is significantly compromised, leading to an accumulation of carcinogenic mutations that heighten the risk of developing bladder cancer (Rafnar et al., 2009). Numerous studies have established a correlation between polymorphisms in the ERCC2 gene and susceptibility to bladder cancer. Specifically, single nucleotide polymorphisms (SNPs) at the ERCC2 rs13181 and rs1799793 loci have been significantly associated with an increased risk of bladder cancer (Rouissi et al., 2011). These SNPs may affect the structural and functional integrity of the ERCC2 protein, ultimately diminishing its effectiveness in repairing DNA damage (Rouissi et al., 2011). Likewise, the XPC gene plays a crucial role in the NER pathway, encoding a protein essential for the recognition of damaged DNA. Mutations or polymorphisms in the XPC gene may impair its ability to detect DNA damage, thereby increasing the sensitivity of bladder epithelial cells to environmental carcinogens. Several studies have shown that the XPC rs2228001 (Lys939Gln) polymorphism is significantly associated with bladder cancer risk, particularly in individuals with occupational exposure to chemical carcinogens (Dou et al., 2013; He et al., 2013). However, some meta-analyses report weak or null associations in non-Asian populations, suggesting gene-environment interactions require further elucidation (Dou et al., 2013; He et al., 2013).

## 4 Metabolism of carcinogens

**Figure 2:**
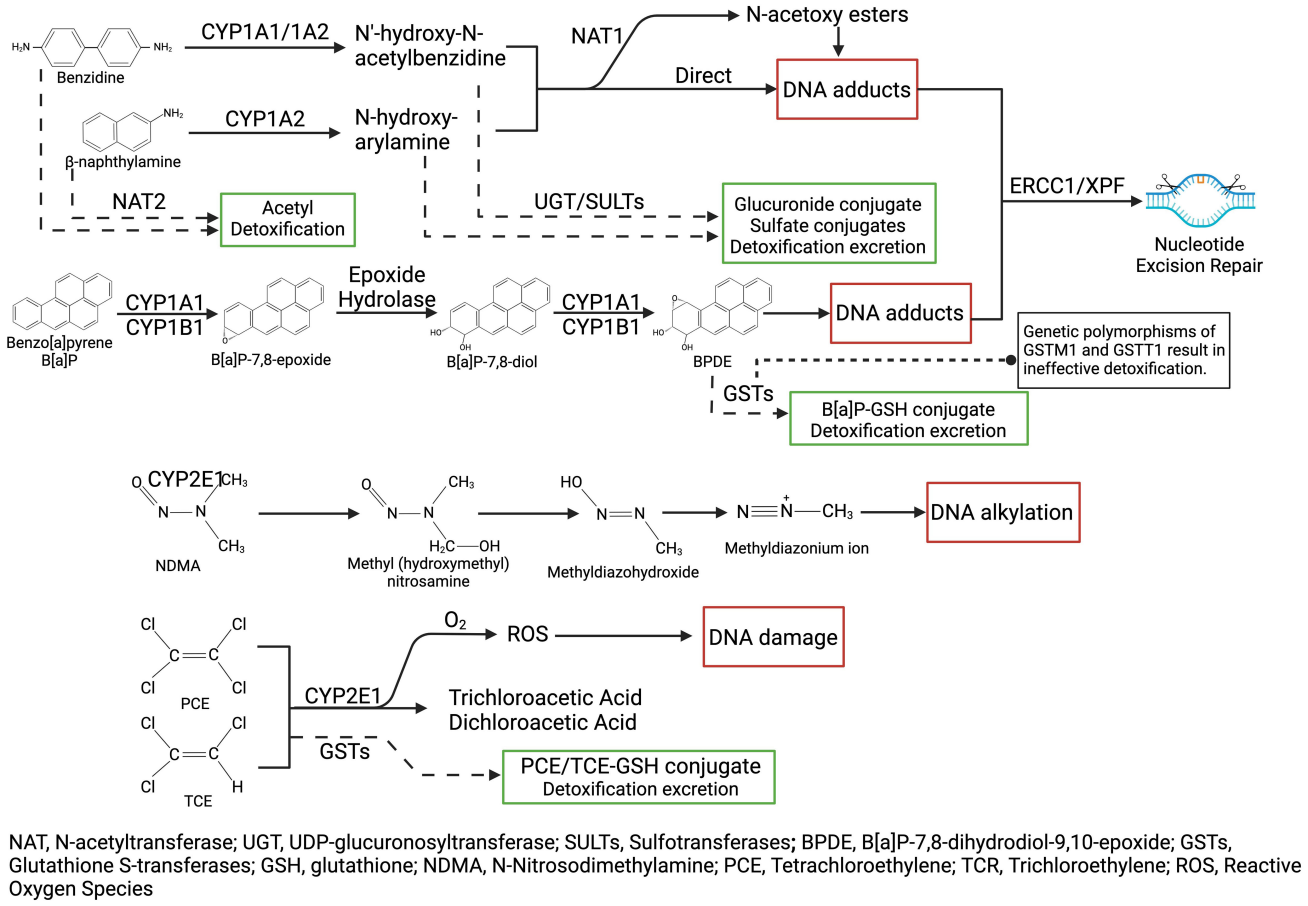
Metabolic activation and detoxification pathways of bladder carcinogens, showing how compounds like aromatic amines and nitrosamines undergo bioactivation, leading to DNA adduct formation, alkylation, and oxidative damage.

The mechanisms underlying bladder cancer are complex, involving the metabolism and detoxification of various environmental and occupational carcinogens. The liver, bladder, and gut microbiota play critical roles in the initial metabolism, secondary activation, and final excretion of these compounds. Key enzyme systems, including the CYP450 family, NAT1/2, UDP-glucuronosyltransferases (UGT), and glutathione S-transferases (GST) families, transform exogenous carcinogens into either potentially carcinogenic or detoxified metabolites through a series of oxidation, reduction, and conjugation reactions. The formation of adducts between these carcinogenic metabolites and DNA in bladder epithelial cells can lead to gene mutations and cellular transformations, significant mechanisms in the development of bladder cancer (**Figure 2**). Integrating recent findings, CYP450 polymorphisms show variable impacts across studies, with stronger effects in high-exposure groups but conflicting results in low-exposure cohorts (Hein, 2002; Thier et al., 2003; Plöttner et al., 2016).

### 4.1 Liver: initial metabolism and activation of carcinogens

The liver is central to the metabolism of bladder carcinogens, converting various exogenous compounds into active or detoxified metabolites through enzyme-catalyzed reactions. For instance, in the metabolism of aromatic amines such as 2-naphthylamine and 4-aminobiphenyl, the CYP450 enzyme family—particularly CYP1A2—catalyzes N-hydroxylation, producing N-hydroxy aromatic amines. These compounds are further converted into highly reactive intermediates by NATs. After being excreted in urine, these intermediates can accumulate in the bladder, ultimately leading to carcinogenesis (Badawi et al., 1995; Hein, 2002). Additionally, PAHs, such as benzo[a]pyrene, are oxidized in the liver by CYP1A1 and CYP1B1 to form epoxides, which are subsequently hydrolyzed by epoxide hydrolase (EH) into highly reactive diol epoxides (e.g., BPDE), thereby driving the molecular mechanisms of bladder cancer (Thier et al., 2003; Plöttner et al., 2016). Nitrosamines, such as NDMA, also depend on hepatic metabolic activation; through the action of CYP2E1, NDMA generates m6G, which possesses alkylating potential (Weltman et al., 1996; García-García et al., 2024). This product can lead to DNA base pairing errors, particularly in the absence of the DNA repair enzyme O6-methylguanine-DNA methyltransferase (MGMT) significantly increasing cancer risk (Armijo et al., 2023; Tessmer and Margison, 2023). Moreover, the liver metabolizes common organochlorines encountered in occupational exposures, such as PCE and TCE. These compounds are converted by CYP2E1 into active products like trichloroacetic acid, which increases the risk of bladder cancer through the induction of ROS (Wu and Berger, 2007; Toyooka et al., 2018). In summary, the complex interplay of various CYP450 enzymes and transferases in the liver metabolizes multiple bladder carcinogens into highly carcinogenic intermediates, underscoring its crucial role in carcinogen metabolism.

The liver also plays a vital role in the detoxification of bladder carcinogens, transforming aromatic amines, PAHs, nitrosamines, arsenicals, organochlorines, and organic solvents into water-soluble products, facilitating their excretion and reducing toxicity. After initial activation by the CYP450 family, aromatic amines (e.g., 2-naphthylamine and 4-aminobiphenyl) undergo conjugation with glucuronic acid or sulfate via UGT and sulfotransferases (SULT), resulting in more easily excretable water-soluble products (Hein, 2002; Uno et al., 2022; Kastrinou Lampou et al., 2023). The metabolism of PAHs, such as benzo[a]pyrene, predominantly relies on GSTs, which conjugate the highly carcinogenic epoxides with glutathione, producing water-soluble metabolites that are excreted via bile or urine, thereby reducing accumulation in target organs and the risk of DNA damage (Alexandrie et al., 2000; Moorthy et al., 2015; Cheng et al., 2022). Organochlorines like PCE and TCE can also be converted by GSTs into water-soluble metabolites bound to glutathione, facilitating their elimination from the body (Moore et al., 2010; Zhang and Liu, 2011; Zhang et al., 2013; Lash et al., 2014). Furthermore, nitrosamines (e.g., NDMA) generate highly alkylating products like m6G through CYP2E1 metabolism, which the liver further processes via UGT and GSTs to minimize their carcinogenic potential (Pegg, 2011; Tessmer and Margison, 2023). In conclusion, the coordinated action of CYP450, UGT, SULT, and GST enzyme systems in the liver effectively manages the metabolism of various bladder carcinogens, significantly reducing their accumulation and carcinogenic potential. This detoxification mechanism is crucial for bladder cancer prevention.

### 4.2 Bladder: secondary activation and action of carcinogenic metabolites

In the bladder, the accumulation of carcinogenic metabolites and their local metabolic processes play a critical role in the development of bladder cancer, featuring mechanisms distinct from those in the liver. The bladder serves not only as the primary excretion route for liver-metabolized carcinogens but also as a site where epithelial cells can further activate these metabolites, facilitating their interaction with DNA and inducing mutations. In the metabolism of aromatic amines, N-hydroxy aromatic amine metabolites generated in the liver enter the bladder via urine and are further acetylated by NAT1 in the bladder epithelial cells, resulting in the production of highly reactive intermediates. These intermediates can directly bind to the DNA of bladder epithelial cells, forming adducts that lead to mutations and activate oncogenes, marking a crucial step in bladder carcinogenesis (Covolo et al., 2008; Dhaini et al., 2018). Regarding PAHs, while glucuronidation by UGT enhances their solubility for excretion, some incompletely metabolized PAH products may persist and bind to bladder DNA, forming adducts that increase the risk of mutations and the development of bladder cancer (Bock et al., 1999; Girard et al., 2008). Additionally, nitrosamine metabolites can accumulate in the bladder and cause alkylation damage to bladder epithelial DNA, further heightening the risk of bladder cancer (Pegg, 2011; Tessmer and Margison, 2023).

## 5 Gut microbiota

The gut microbiota is the largest microbial community in the human body, with bacterial cells vastly outnumbering human cells. In a typical adult, the gut microbiota contains approximately 3.8 × 10¹³ bacterial cells—about ten times the number of human cells—and its genetic material exceeds the human genome by 30-fold, highlighting its immense metabolic potential (Li et al., 2014; Sender et al., 2016). Beyond food digestion, gut bacteria play vital roles in detoxification, endocrine function, neurotransmission, immune regulation, and pathogen defense (Kamada et al., 2013; Fung et al., 2017; Fan and Pedersen, 2021). Through their interaction with the host, metabolites such as short-chain fatty acids (e.g., acetate, butyrate), amino acids, and bile acids significantly influence host nutrition, immune responses, and gut barrier integrity, while also regulating systemic metabolism (Morrison and Preston, 2016). Consequently, the gut microbiota is a key “modulator” of both local and systemic metabolism, ensuring proper physiological function. The human gut microbiota is a dynamic ecosystem composed of 400–1000 bacterial species, mainly from two dominant phyla: *Firmicutes* and *Bacteroidetes*. The microbiota’s composition remains relatively stable in adulthood, with microbial diversity established early in life and shaped by diet and immune system maturation (Qin et al., 2010). Its balance is influenced by environmental, dietary, pharmacological, and lifestyle factors, resulting in changes over time (Rothschild et al., 2018; Kolodziejczyk et al., 2019; Hughes et al., 2020; Han et al., 2021; Chen et al., 2022). Dysbiosis—an imbalance in microbial composition—can lead to the overgrowth of harmful bacteria or a reduction in beneficial species, contributing to chronic inflammation, immune dysfunction, and an increased risk of diseases. Growing evidence links these changes to conditions such as obesity, type 2 diabetes, cardiovascular diseases, and cancer (Essex et al., 2024; Zhang et al., 2024). In summary, the gut microbiota is essential for maintaining health and metabolic homeostasis, with its balance playing a critical role in preventing and managing chronic diseases and cancer.

### 5.1 Gut microbiota and bladder cancer

The relationship between gut microbiota and bladder cancer has garnered increasing attention in recent years. Numerous studies suggest that gut dysbiosis is closely linked to the onset and progression of bladder cancer. For instance, Mendelian randomization (MR) analysis has revealed that specific bacteria, such as *Bifidobacterium*, *Actinobacteria*, and certain gut residents (e.g., *Ruminococcus torques group*), are associated with an increased risk of bladder cancer (Mingdong et al., 2023). Conversely, species like *Allisonella* exhibit an inverse correlation with cancer risk (Mingdong et al., 2023). Bladder cancer patients show significant differences in their gut microbiota compared to healthy controls. Beneficial bacteria, such as *Prevotella*, are notably reduced (He et al., 2020). Moreover, the concentration of short-chain fatty acids (SCFAs), particularly butyric acid, is significantly lower in these patients (He et al., 2020). Furthermore, gut microbes like *Parabacteroides distasonis* may enhance anti-tumor immunity by promoting CD4+ and CD8+ T cell activation, potentially boosting the efficacy of immunotherapy (Wang et al., 2024). Specific species, including *Lactobacillus casei Shirota* and *Bifidobacterium breve*, can modulate the metabolism of chemotherapy drugs or influence intestinal immune cells, altering treatment outcomes (Miyake et al., 2023). These bacteria can affect drug concentrations and, by shaping the intestinal immune environment, either enhance or diminish the effectiveness of chemotherapy. These findings highlight the crucial role of the gut microbiota in bladder cancer pathogenesis, progression, and therapy. They suggest potential microbiome-based diagnostic and therapeutic strategies for bladder cancer (Yang et al., 2024).

Human observational studies further support these associations. For example, a 2024 study of 142 urothelial carcinoma patients analyzed fecal microbiomes and linked specific profiles to treatment responses (Bukavina et al., 2024). Another observational cohort in China showed reduced *Prevotella* and SCFAs in bladder cancer patients compared to controls (He et al., 2020). Emerging evidence also highlights the gut-bladder axis, where gut dysbiosis influences urinary microbiota; for instance, a 2025 study identified urinary dysbiosis patterns in primary versus recurrent bladder cancer correlating with recurrence risk, potentially driven by gut-derived metabolites (Butt and De Biase, 2025; Sheng et al., 2025). However, these studies often involve small cohorts (n<200), limiting generalizability, and are confounded by factors like diet, age, smoking, and antibiotics, which can alter microbiota independently of cancer (Pallares-Mendez et al., 2024; Lang et al., 2025). Larger, prospective human studies are needed to disentangle these effects. Recent Mendelian randomization analyses (2023–2025) provide causal insights, identifying Bilophila as a potential pathogenic initiator in bladder cancer via gut dysbiosis, though replication in diverse populations is required (Yang et al., 2024). Additionally, a 2025 murine model study on upper tract urothelial carcinoma (UTUC)—a malignancy biologically related to bladder cancer—demonstrated that dietary interventions reshaped the gut microbiota, upregulating Parabacteroides distasonis and suppressing carcinogenesis (Yamamoto et al., 2025). Given the shared etiological and mechanistic pathways (e.g., carcinogen metabolism in urothelial tissues), this suggests similar microbiome modulations could apply to bladder cancer prevention, though human validation is pending.

**Figure 3:**
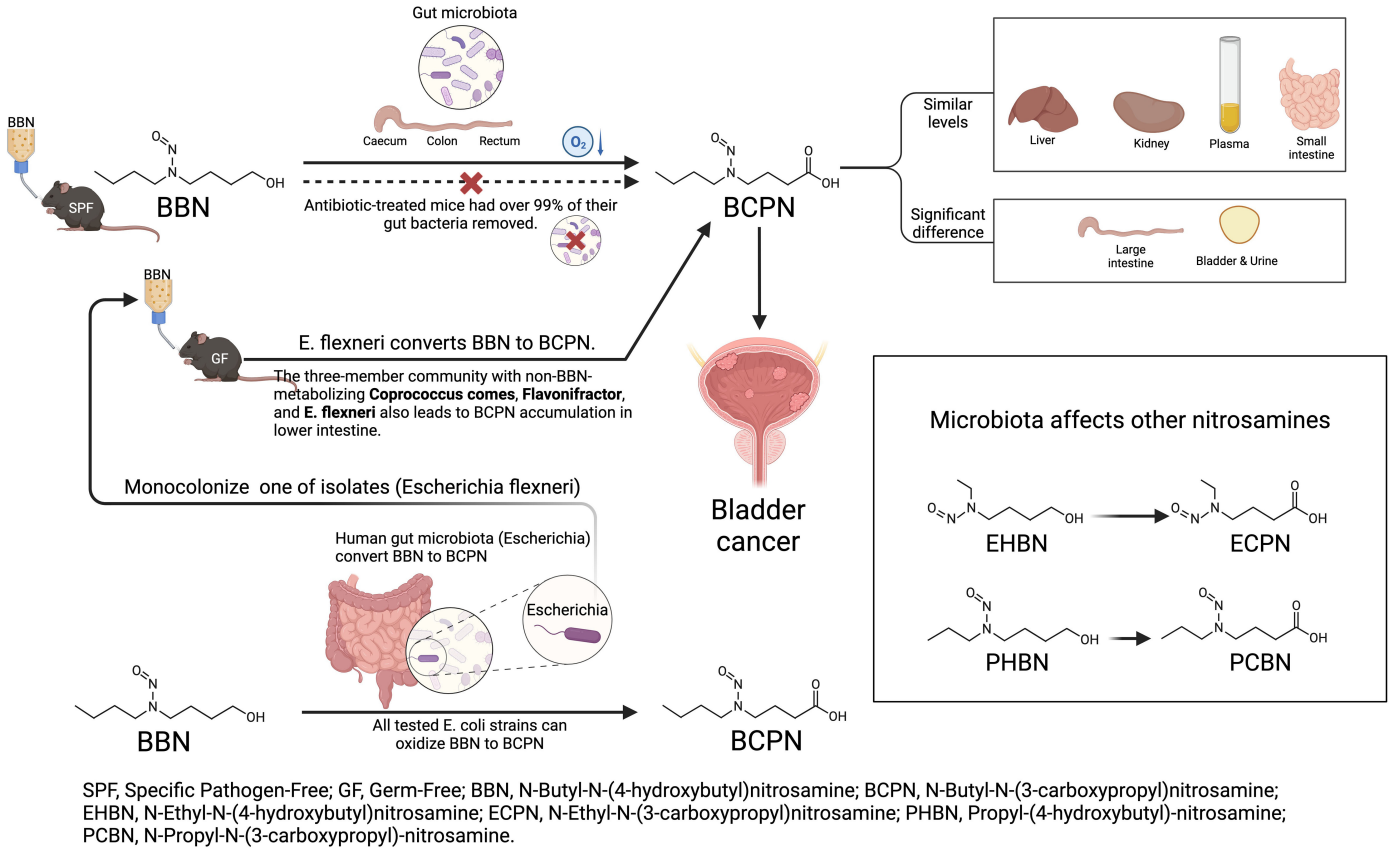
The schematic illustrates the gut microbiome-driven conversion of BBN to BCPN, emphasizing the role of *E. coli* in metabolic activation and its potential link to bladder cancer risk. This pathway further highlights the broader impact of microbial activity on nitrosamine metabolism, as demonstrated with other nitrosamine derivatives.

The gut microbiota plays a significant role in the metabolism of carcinogens related to bladder cancer, influencing the metabolic processes and carcinogenic potential of these compounds through both activation and detoxification pathways. Certain gut microbes can biotransform aromatic amines and nitrosamines via specific enzymatic mechanisms, generating highly reactive intermediates that may cause localized damage in the intestine. These metabolically active products can also enter systemic circulation, impacting distant organs such as the bladder and potentially increasing cancer risk. A pivotal study elucidated the mechanism by which gut microbiota contribute to the metabolism of carcinogens in distal organs. This research demonstrated that gut microbiota could convert N-butyl-N-(4-hydroxybutyl) nitrosamine (BBN) into the more active metabolite N-butyl-(3-hydroxybutyl) pyridine (BCPN), which acts on bladder tissue and significantly promotes bladder cancer development (**Figure 3**). This finding highlights the indirect regulatory role of gut microbiota in the pathogenesis of bladder cancer, suggesting that modulating gut microbial ecology may serve as a potential preventive intervention (Lloyd, 2024; Mani, 2024; Roje et al., 2024). Conversely, certain gut microbiota exhibit detoxification potential in the metabolism of specific carcinogens. Evidence from drug metabolism studies indicates that some microbial communities can modulate the toxicity and activity of certain compounds through synergistic interactions with the host, thereby reducing their harmful effects (Haiser and Turnbaugh, 2012). Critically, while animal models support activation pathways, human studies show mixed results; for instance, microbiota depletion reduces tumor risk in mice but observational data in humans are confounded by lifestyle factors (Roje et al., 2024; Yang et al., 2024).

### 5.2 Gut microbiota metabolism of carcinogens

**Figure 4:**
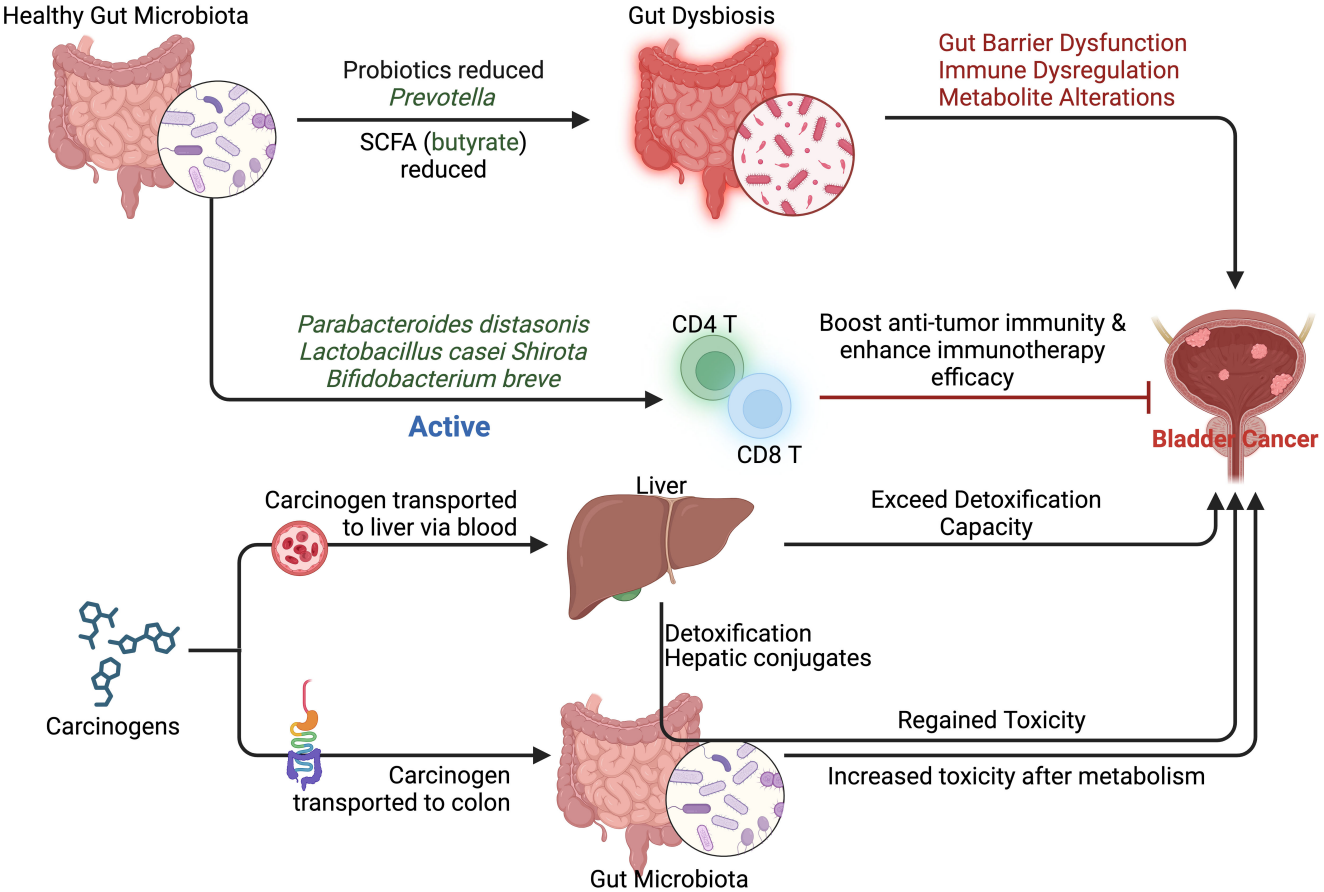
The relationship between gut microbiota and bladder cancer: Dysbiosis, induced metabolic changes, immune regulation, and carcinogenic metabolites all potentially contribute to the initiation and progression of bladder cancer.

The gut microbiota plays a pivotal role in the metabolism of exogenous substances. As early as 1973, Scheline suggested that its metabolic capacity might rival that of the liver (Scheline, 1973). Subsequent studies confirmed that the gut microbiota can perform diverse chemical transformations of drugs and chemicals, including reduction, hydrolysis, decarboxylation, and dehydrogenation reactions (Haiser and Turnbaugh, 2013). This discovery provided key insights into the microbiota’s metabolic functions within the human body. However, its role in metabolizing environmental chemicals, particularly carcinogens, remains underexplored. The gut microbiota significantly impacts the transformation of exogenous substances, especially through processes such as deconjugation and reduction, which convert larger compounds into smaller, non-polar molecules more easily reabsorbed by the body. This affects their distribution and toxicity (Haschek-Hock et al., 2022). Notably, the microbiota metabolizes carcinogens through enzymes like azo reductase, nitro reductase, β-glucuronidase, sulfatase, and β-cleavage enzymes (Williams et al., 1970; Rafii et al., 1990; Rafil et al., 1991; Roldán et al., 2008). Polycyclic aromatic hydrocarbons (PAHs), such as benzo(a)pyrene and phenanthrene, exhibit estrogenic activity after gut microbiota metabolism, suggesting that gut bacteria may enhance their carcinogenic effects by converting PAHs into estrogenic compounds (Van de Wiele et al., 2005). Both murine and human microbiota can also regenerate benzo(a)pyrene from its hepatic conjugates, potentially reversing endogenous detoxification and posing toxicological risks (Renwick and Drasar, 1976). Additionally, nitro-PAHs and nitro-toluenes, upon reduction by the gut microbiota, produce mutagenic and carcinogenic metabolites, further amplifying their carcinogenic potential (Doolittle et al., 1983; Möller et al., 1988; Möller, 1994). The gut microbiota also plays a crucial role in the metabolism of other environmental pollutants, such as pesticides and metals. For instance, organochlorine pesticides like DDT are converted into more toxic metabolites (Kim et al., 2014), while metals like mercury and arsenic undergo redox reactions, altering their toxicity and accumulation in the body (Nakamura et al., 1977; Rowland and Davies, 1981). Azo dyes, such as Sudan dyes and Para Red, are metabolized by gut bacteria into carcinogenic aromatic amines, increasing cancer risk (Macholz et al., 1985; Xu et al., 2010). These findings underscore the gut microbiota’s essential role in modulating the metabolism and toxicity of environmental chemicals (**Figure 4**), particularly carcinogens (**Supplementary Table 1**).

Regarding nitrosamine metabolism, human-relevant species include E. coli, which converts BBN to BCPN in models, increasing bladder tumor risk (Mani, 2024; Roje et al., 2024). Observational data link dysbiosis (e.g., overgrowth of *Proteobacteria*) to higher nitrosamine levels in bladder cancer patients. However, evidence is limited by low microbial biomass in samples and potential contamination, with conflicting reports on whether certain bacteria (e.g., *Bilophila*) promote or mitigate nitrosamine effects (Isali et al., 2024; Yang et al., 2024). Recent 2024 studies emphasize that microbiota depletion alters nitrosamine toxicokinetics in humans, but larger cohorts are needed to confirm species-specific roles.

## 6 Other carcinogenic factors

The development of bladder cancer is a complex, multifactorial process influenced by an interplay of genetic, environmental, lifestyle, dietary, and hormonal factors. Lifestyle modifications, such as increasing fluid intake and improving dietary habits, can significantly reduce the risk of bladder cancer. Additionally, the impact of sex differences and the role of androgen receptors in the pathogenesis of bladder cancer are critical considerations. A comprehensive understanding of these factors is essential for elucidating the mechanisms underlying bladder cancer, offering new perspectives for prevention and treatment strategies.

### 6.1 Lifestyle and dietary factors

Bladder cancer risk is affected not only by genetic predispositions and environmental exposures but also by lifestyle choices and dietary habits. Insufficient fluid intake is recognized as an independent risk factor for bladder cancer. Reduced fluid consumption leads to concentrated urine, which extends the exposure of bladder epithelial cells to carcinogenic substances, thereby increasing the risk of DNA damage and mutations (Michaud et al., 1999; Bar David et al., 2004). Conversely, adequate fluid intake dilutes harmful metabolites in urine and facilitates their rapid excretion, reducing carcinogenic exposure to bladder epithelial cells (Di Maso et al., 2016). Extensive prospective cohort studies have shown a negative correlation between daily fluid intake and bladder cancer incidence, particularly among high-risk groups such as long-term smokers (Ros et al., 2011). Furthermore, specific dietary carcinogens are strongly associated with bladder cancer risk. Low levels of N-nitrosamines may be present in certain foods, where they are absorbed through the gastrointestinal tract into the bloodstream, metabolized by the liver, and subsequently excreted into the bladder, increasing the carcinogenic risk to bladder epithelial cells (Mirvish, 1995). Notably, dietary exposure to N-nitrosamines correlates positively with bladder cancer risk, especially with the consumption of processed meats and foods cooked at high temperatures, which significantly enhance the formation of these compounds (Ward et al., 1997). Consequently, lifestyle interventions, such as increasing fluid intake and avoiding foods containing potential carcinogens, are crucial strategies for preventing bladder cancer.

### 6.2 Hormonal factors

The incidence of bladder cancer is significantly higher in men than in women, potentially due to the influence of androgens and their receptors (androgen receptors, AR) in the carcinogenic process. Androgens can regulate the expression of various CYP450 enzymes, such as CYP1A1, CYP1A2, and CYP1B1, through the AR signaling pathway. These enzymes play crucial roles in the oxidative metabolism of aromatic amines and PAHs, thereby accelerating the metabolic activation of carcinogens in the bladder (Kelly and Jones, 2013). In addition, enzymes such as CYP3A4 and CYP2E1 are involved in the metabolism of PAHs and nitrosamines, with CYP3A4 expression being significantly regulated by androgens. CYP2E1 may also contribute to the activation of carcinogens under androgen influence (Gorski et al., 2000). This suggests that androgens, by modulating the expression of CYP450 enzymes, can impact the metabolic activity and toxicity of carcinogens, thereby increasing an individual’s risk of bladder cancer. Furthermore, androgens may downregulate the expression of UGTs, such as UGT1A, which diminishes the detoxification capacity of bladder epithelial cells. UGTs are important phase II metabolic enzymes that convert carcinogens into soluble forms for excretion, reducing their activity. However, androgens inhibit the expression of these enzymes through androgen receptor (AR) signaling, potentially increasing the toxic effects of carcinogens within bladder cells (Izumi et al., 2013; Sundi et al., 2024). Thus, androgens may significantly influence bladder carcinogenesis by regulating various metabolic enzymes and affecting detoxification pathways, ultimately impacting an individual’s carcinogenic risk.

## 7 Carcinogen and immune microenvironment

**Figure 5:**
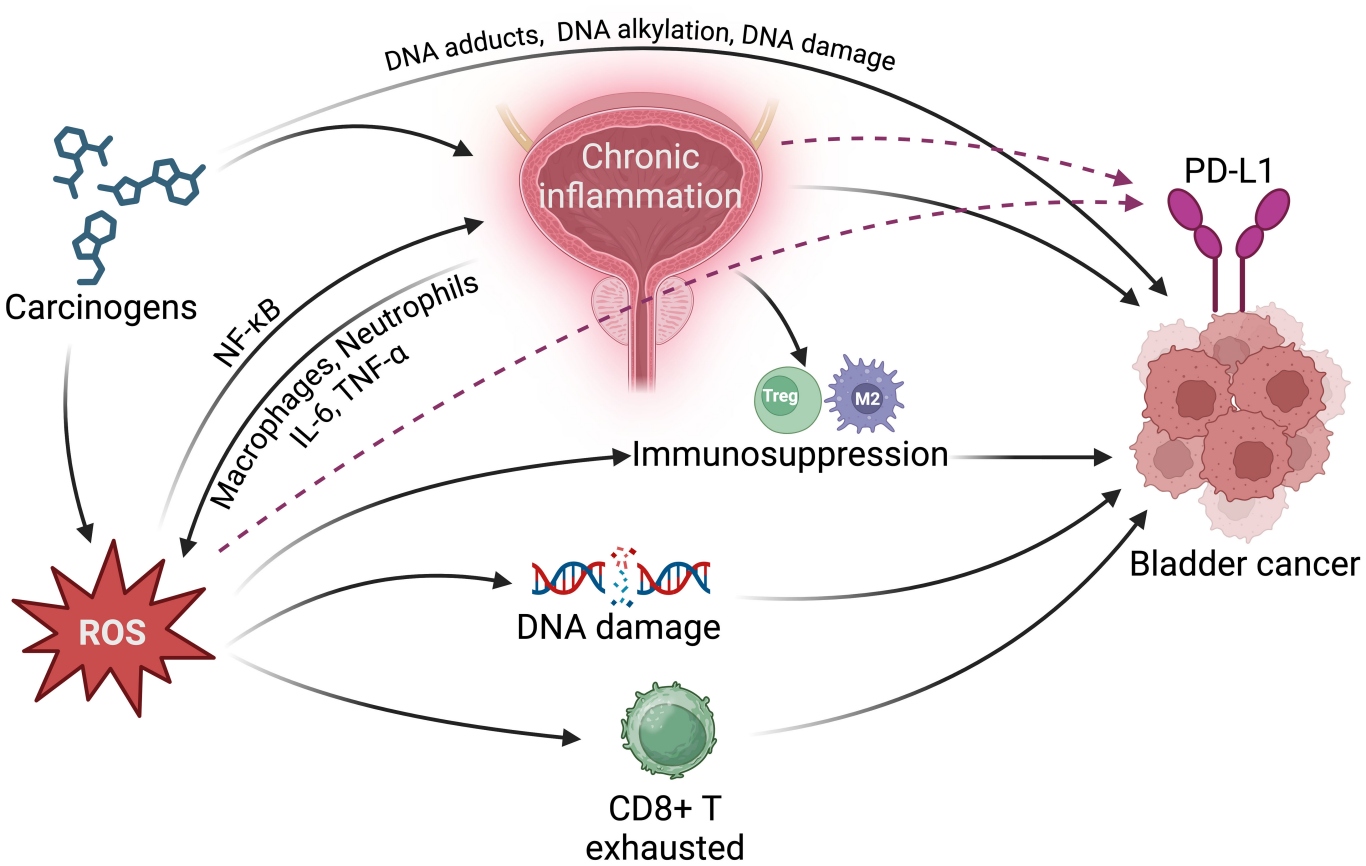
Carcinogens promote the development of bladder cancer by forming a microenvironment of chronic inflammation and reactive oxygen species (ROS). The production of ROS activates NF-κB to release proinflammatory cytokines, and macrophages and neutrophils in chronic inflammation release cytokines to promote the production of ROS. ROS promotion induces DNA damage, recruits immunosuppressive cells, and leads to CD8+ T cell exhaustion. The release of ROS and the chronic inflammatory environment jointly promote the progression of bladder cancer, and the upregulation of PD-L1 by tumor cells further enhances the immune escape mechanism.

Prolonged exposure to external carcinogens can induce significant alterations in the bladder microenvironment, particularly with substances that are excreted through urine and maintain prolonged contact with bladder epithelial cells, such as aromatic amines, arsenic, and nitrosamines (Fernández et al., 2019; Lobo et al., 2022). These carcinogens modify the TME through various biological mechanisms, resulting in persistent chronic inflammation, increased oxidative stress, and enhanced immune suppression. These changes not only facilitate tumor initiation and progression but also impair anti-tumor immune responses (**Figure 5**). Recent studies link these effects to microbiota dysbiosis, but human data are limited by confounders.

### 7.1 Carcinogen-induced chronic inflammation

Chronic inflammation in the bladder significantly contributes to the development of bladder cancer. Prolonged inflammation accelerates carcinogenesis in bladder epithelial cells through multiple mechanisms. First, inflammation leads to the excessive production of ROS and nitrogen radicals, causing direct DNA damage and promoting the accumulation of mutations (Wigner et al., 2021). Inflammatory mediators, such as cytokines and chemokines, also promote cell proliferation and inhibit apoptosis, creating a microenvironment that favors carcinogen activity (Wigner et al., 2021). Additionally, chronic inflammation alters the bladder’s metabolic environment, prolonging the retention of carcinogenic substances and intensifying their damaging effects on epithelial cells (Babjuk et al., 2022). Inflammatory responses can further inhibit DNA repair mechanisms, speeding up the onset of bladder cancer. This interplay between inflammation and the bladder environment underscores the potential value of targeting inflammation in bladder cancer prevention and treatment.

Chronic inflammation also indirectly amplifies the activation of carcinogens. During inflammation, macrophages and neutrophils in the bladder release ROS, which activate CYP450 enzymes (e.g., CYP1A1 and CYP1B1), enhancing the conversion of aromatic amines and PAHs into more carcinogenic metabolites (Shimada and Fujii-Kuriyama, 2004; Stading et al., 2020; Li et al., 2024). Inflammation-induced accumulation of nitrites contributes to the formation of nitrosamines. High nitric oxide (NO) levels promote N-nitrosation reactions, leading to the generation of nitrosamine carcinogens (Bartsch and Montesano, 1984; Vikram et al., 2024). Chronic inflammation and bladder infections further increase nitrosamine production, which has been linked to a higher incidence of bladder cancer in both animal studies and epidemiological research (Hicks et al., 1982; Kustrimovic et al., 2024). Moreover, inflammation-induced metabolic changes prolong carcinogen retention, increasing the exposure of bladder epithelial cells to harmful substances and raising the risk of bladder cancer (Keimling et al., 2014). To avoid redundancy, note that ROS-mediated damage is detailed in Section 3.3; here, we emphasize its role in inflammation amplification.

Carcinogens foster a microenvironment conducive to cancer development by inducing chronic inflammation in bladder tissues. Chronic inflammation is a critical factor in bladder cancer pathogenesis. Continuous exposure to carcinogens triggers bladder epithelial cells and infiltrating immune cells (such as macrophages and neutrophils) to release pro-inflammatory cytokines (e.g., IL-6, TNF-α, NF-κB), which amplify the inflammatory response and disrupt normal tissue structure and function (Balkwill and Mantovani, 2001; Yan et al., 2006; Zappavigna et al., 2020). NF-κB, a key transcription factor, regulates genes involved in inflammation and apoptosis, driving tumor progression (Karin and Greten, 2005; Sun, 2017; Taniguchi and Karin, 2018). This persistent inflammation exposes bladder epithelial cells to repeated cycles of damage and repair, creating conditions that favor DNA mutations and abnormal cell proliferation.

In the context of chronic inflammation, elevated ROS levels in the TME result from both metabolic dysregulation in epithelial cells and ROS release by immune cells during inflammatory responses (Mittal et al., 2014; Tseng et al., 2017). ROS directly damage bladder epithelial cell DNA, causing base oxidation and DNA strand breaks, which lead to gene mutations and chromosomal abnormalities (Reuter et al., 2010; Caliri et al., 2021). Furthermore, ROS activate cancer-related signaling pathways, including Mitogen-Activated Protein Kinase (MAPK) and NF-κB, promoting tumor cell proliferation and resistance to apoptosis (Lee et al., 2017; Hamidi and Ivaska, 2018; Khan et al., 2023). In summary, chronic inflammation plays a crucial role in both activating carcinogens and promoting cancer progression, highlighting the importance of targeting inflammation in bladder cancer prevention and treatment. Conflicting evidence from 2024 studies suggests NF-κB inhibition may vary by tumor stage, with potential off-target effects in clinical trials (Karin and Greten, 2005; Sun, 2017; Taniguchi and Karin, 2018).

### 7.2 Immune suppression and immune evasion

The immune suppression and evasion triggered by carcinogens and chronic inflammation within the TME are critical factors in tumor development and invasion. Within this environment, ROS contribute to tumor progression by inducing phenotypic changes in macrophages. For instance, ROS can promote the polarization of macrophages into the M2 phenotype, which secretes anti-inflammatory cytokines such as Interleukin-10 (IL-10), thereby inhibiting normal immune responses and facilitating tumor cells in evading immune surveillance (Tsai et al., 2021; Liu et al., 2022; Li et al., 2024). Additionally, ROS in the TME can indirectly enhance the immunosuppressive capacity of regulatory T cells (Treg) by inducing their apoptosis, which leads to the release of adenosine and further increases immune suppression (Maj et al., 2017). Chronic exposure to ROS also contributes to the functional decline of T cells, particularly CD8+ T cells, exacerbating immune exhaustion and further diminishing the capacity for tumor clearance (Pritykin et al., 2021; Scharping et al., 2021; Chen et al., 2022; Wu et al., 2023). Moreover, oxidative stress and inflammation induced by carcinogens may upregulate the expression of Programmed Death-Ligand 1(PD-L1) on bladder tumor cells. PD-L1 serves as a critical immune checkpoint molecule that inhibits T cell anti-tumor activity by binding to PD-1 on T cells (Antonangeli et al., 2020; Bailly, 2020; Battaglia et al., 2023). Exposure to specific carcinogens can lead to increased PD-L1 expression, primarily through the activation of the NF-κB signaling pathway (Chen and Mellman, 2017; Zheng et al., 2023).

## 8 Foods and drugs aiding in detoxifying carcinogens

High-risk carcinogens for bladder cancer, including aromatic amines, PAHs, nitrosamines, arsenic, and organochlorine compounds, primarily exert their carcinogenic effects through metabolic activation into toxic intermediates, which subsequently cause DNA damage or mutations. Certain foods and drugs, however, can significantly mitigate these risks by modulating carcinogen metabolism, reducing oxidative stress, and enhancing DNA repair (Zhang and Talalay, 1994; Lambert and Elias, 2010). By decreasing the formation of toxic metabolites, accelerating their clearance, minimizing oxidative damage, and strengthening DNA repair mechanisms, these interventions collectively reduce the carcinogenic impact of these compounds on bladder cells. This integrated mechanism provides a scientific foundation for bladder cancer prevention, underscoring the importance of dietary and pharmacological interventions, particularly in high-risk populations. However, evidence is mixed; while preclinical studies show promise, human trials often report modest effects due to dose variability, individual genetic differences, and potential interactions with other factors like smoking (Zhang and Talalay, 1994; Lambert and Elias, 2010). Some interventions may even have adverse effects at high doses, and long-term efficacy remains unproven in large cohorts.

Many foods help reduce carcinogen-induced toxicity by modulating the activity of metabolic enzymes. Cruciferous vegetables, such as broccoli, cauliflower, and Brussels sprouts, are rich in glucosinolates, which are hydrolyzed into isothiocyanates (ITCs) upon consumption. ITCs have been shown to activate phase II detoxifying enzymes like glutathione S-transferase (GST) through the Nrf2/ARE pathway. This induction enhances the clearance of reactive metabolites, such as those derived from aromatic amines and PAHs, thereby reducing their toxic effects (Boddupalli et al., 2012; Bayat Mokhtari et al., 2018). Additionally, catechins found in green tea inhibit CYP450 enzyme activity, effectively lowering the formation of toxic intermediates from nitrosamines and PAHs during metabolic activation (Boots et al., 2008; Lambert and Elias, 2010). Fruits and vegetables rich in quercetin, such as onions and apples, also exhibit antioxidant properties that scavenge ROS and inhibit carcinogen activation (Głąbska et al., 2020; Hardt et al., 2022). Nevertheless, epidemiological studies yield conflicting results; for example, while some cohort studies link high vegetable intake to reduced bladder cancer risk, others find no association after adjusting for confounders like smoking (Hardt et al., 2022). Human bioavailability of these compounds is low, and benefits may be limited to specific genotypes.

Vitamins and minerals, such as vitamin E and selenium, can enhance antioxidant defenses and DNA repair. However, large-scale trials like SELECT (2011) showed no preventive effect for selenium and vitamin E in prostate cancer, with potential risks at high doses (Klein et al., 2011; Vinceti et al., 2018). Dietary fiber from whole grains promotes gut health and may bind carcinogens, reducing absorption (Yu et al., 2020). Phytochemicals in spices like turmeric (curcumin) inhibit NF-κB and ROS, but clinical evidence is preliminary (Bacon et al., 2003; Al-Ishaq et al., 2020). Sulforaphane from broccoli and quercetin from fruits modulate PhIP-DNA adducts in models (Bacon et al., 2003). Overall, while these foods show mechanistic promise, real-world benefits are dose-dependent and may not translate uniformly across populations; randomized trials are needed to balance optimism with evidence (Salech et al., 2020; Navas and Carnero, 2021).

## 9 Future perspectives on bladder cancer control: from carcinogen control to innovative preventive strategies

With deeper insights into the mechanisms of bladder cancer carcinogens, future efforts to control this cancer should not only focus on traditional treatments but also expand to include prevention of carcinogen exposure, metabolic regulation, and early intervention. By integrating environmental, dietary, pharmaceutical, and public health measures, a comprehensive approach to bladder cancer prevention and early treatment can be achieved. With continuous advancements in science, particularly in genomics, nutrition, and drug development, bladder cancer control is poised to reach new heights, offering more effective solutions for global prevention and treatment.

### 9.1 Precision carcinogen monitoring and control: from early detection to effective intervention

Firstly, precise monitoring and control of carcinogen exposure will be key to future bladder cancer prevention. With the increasing environmental pollution and occupational exposure, especially in high-risk populations (such as workers exposed to aromatic amines, PAHs, nitrosamines, etc.), real-time monitoring of carcinogen exposure through advanced detection technologies will be crucial in reducing bladder cancer incidence. Future efforts should emphasize the development and application of biomarkers to assess individual carcinogen exposure and its metabolic products through blood or urine tests, providing early warning signals for bladder cancer. This approach will allow for intervention at the initial stages of exposure, significantly reducing bladder cancer risks.

### 9.2 Personalized dietary interventions: tailored nutritional plans for individuals

Next, personalized dietary interventions are emerging as an innovative strategy for bladder cancer prevention. Research has shown that certain foods and compounds can regulate carcinogen metabolism and reduce their carcinogenic potential. However, individual responses to these foods and medications may vary, influenced by genetic diversity, lifestyle, and other factors. Therefore, integrating genomics and nutrition into personalized dietary plans for high-risk populations will greatly enhance intervention effectiveness and reduce bladder cancer incidence. Personalized nutrition should not only focus on food selection but also be tailored based on individual genomic data. Moreover, exploring synergistic effects of foods and drugs, especially combining natural plant compounds (such as sulforaphane, quercetin, and green tea catechins) with traditional medications (like nicotinamide and anti-cancer drugs), could open new pathways for bladder cancer prevention and treatment.

### 9.3 Targeting metabolic enzymes: developing new drugs and precision therapies

In terms of drug development, future research should focus more on creating targeted inhibitors of carcinogen-metabolizing enzymes. Specifically, inhibitors of CYP450 and related enzymes could reduce the activation of carcinogens and lower their carcinogenic potential. Additionally, modulating the Nrf2/ARE pathway to enhance the expression of detoxifying enzymes and antioxidant defenses could be a new avenue for bladder cancer prevention. By combining detoxification enhancement with DNA repair promotion in drug therapies, a more effective and personalized treatment approach for bladder cancer could be developed. This strategy would complement traditional treatments, providing precise inhibition of carcinogen activation, reducing patient treatment burdens, and improving therapeutic outcomes.

### 9.4 Gut microbiota and bladder cancer: the “hidden trap” in carcinogen metabolism

Recently, the role of gut microbiota in cancer prevention has gained increasing attention, particularly in the metabolism of carcinogens in bladder cancer. Certain carcinogens (such as aromatic amines and nitrosamines) undergo metabolic conversion in the gut, where microbiota can transform them into more carcinogenic forms, increasing bladder cancer risk. Gut microbiota interventions are seen as an important strategy for future bladder cancer prevention. By using probiotics, prebiotics, and dietary fibers, we can improve gut microbiota composition, suppress harmful bacterial growth, and reduce the production of harmful metabolites, thus lowering carcinogen transformation in the body. For example, probiotics such as Lactobacillus and Bifidobacterium have been shown to inhibit certain gut bacterial enzymes that produce nitrosamines. Additionally, dietary fiber can improve gut permeability and barrier function, reducing the residence time of carcinogens in the gut and promoting the growth of beneficial microbes, further reducing harmful carcinogen production. Therefore, dietary interventions combined with gut microbiota modulation can serve as an important preventive measure, especially for high-risk individuals exposed to carcinogens over extended periods.

### 9.5 Early intervention and strengthening public health policies: from prevention to long-term control

Finally, early intervention and the strengthening of public health policies will be a long-term strategy to reduce the burden of bladder cancer. For high-risk populations, governments and public health institutions can enhance health education, standardize occupational safety measures, improve air and water quality, and promote cancer-preventing dietary patterns to reduce environmental carcinogen exposure. Moreover, the development of preventive vaccines and drugs could become a significant breakthrough in bladder cancer prevention. For instance, immune checkpoint inhibitors have been successful in treating several cancers, and similar strategies may play a role in bladder cancer immunoprevention. By integrating health policies, environmental monitoring, and public education, we could achieve early identification and prevention of bladder cancer, significantly lowering its global incidence.

## 10 Conclusion

**Figure 6:**
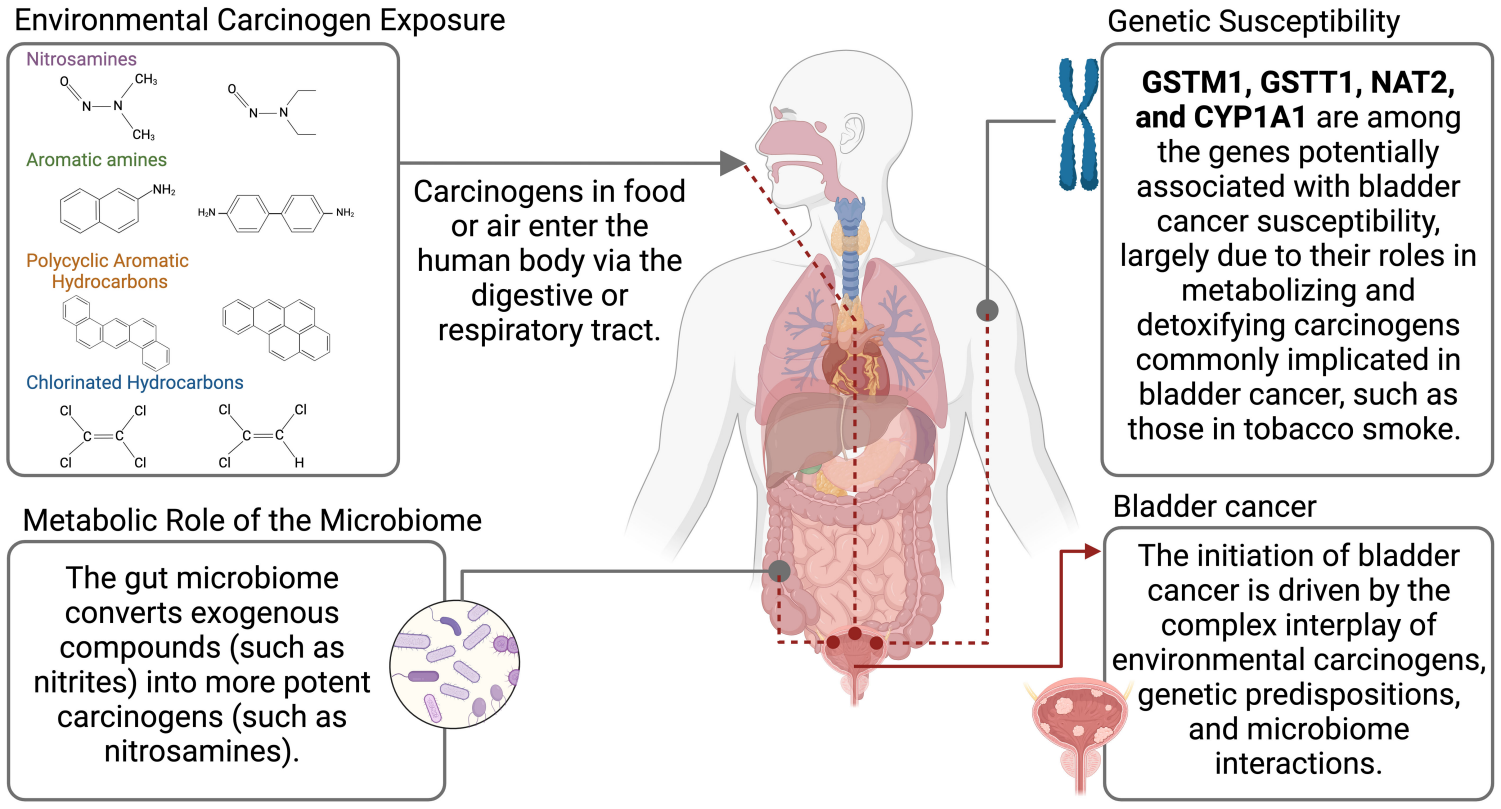
Environmental carcinogens promote the initiation of bladder cancer, influenced by genetic susceptibility and microbiome metabolism, leading to increased DNA damage and elevated cancer risk.

Bladder cancer development results from a complex interplay of environmental carcinogen exposure, genetic susceptibility, and microbial metabolic activity. This review has focused on the carcinogenic mechanisms of environmental exposures and their cumulative biological effects in bladder cancer. Bladder carcinogenesis is initiated by exposure to various environmental carcinogens, including nitrosamines, aromatic amines, PAHs, and chlorinated hydrocarbons. These compounds enter the body through diet, air, or occupational routes, accumulate in the bladder, and interact with epithelial cells over time, leading to DNA damage and a heightened risk of malignant transformation. However, the cancer-promoting effects of these carcinogens are influenced by individual genetic backgrounds. Polymorphisms in specific metabolic genes, such as *GSTM1*, *GSTT1*, *NAT2*, and *CYP1A1*, affect sensitivity to carcinogens. Certain gene variants reduce detoxification capabilities, significantly increasing the risk of bladder cancer, particularly among smokers, in whom gene-environment interactions are especially impactful. The role of the microbiome in bladder carcinogenesis is also crucial. Studies suggest that gut microbiota can convert exogenous compounds like nitrites into potent carcinogens, such as nitrosamines, increasing harmful metabolite accumulation in the bladder. The microbiome’s metabolic functions, in conjunction with genetic and environmental factors, form a complex carcinogenic network that influences bladder cancer susceptibility (**Figure 6**).

In summary, the carcinogenic processes that contribute to bladder cancer involve a multifaceted interaction of environmental exposure, genetic predisposition, and microbial activity. Detailed understanding of how specific environmental carcinogens, such as nitrosamines and aromatic amines, exert their effects at the cellular level will not only clarify bladder cancer pathogenesis but also strengthen primary prevention strategies. This includes targeted monitoring and management of high-risk carcinogens in diet, air pollution, and occupational settings. Furthermore, personalized prevention approaches, such as microbiome modulation or genetic-based interventions, hold promise for tailored strategies against bladder cancer. These insights provide new perspectives on bladder cancer mechanisms and establish a foundation for more effective, targeted public health policies and personalized treatment options, advancing bladder cancer prevention and control toward higher precision and efficacy.

## References

[B1] Abbaoui B. Lucas C. R. Riedl K. M. Clinton S. K. Mortazavi A. (2018). Cruciferous vegetables, isothiocyanates, and bladder cancer prevention. Mol. Nutr. Food Res. 62, e1800079. doi: 10.1002/mnfr.201800079, PMID: 30079608 PMC6196731

[B2] Alexandrie A. K. Warholm M. Carstensen U. Axmon A. Hagmar L. Levin J. O. . (2000). CYP1A1 and GSTM1 polymorphisms affect urinary 1-hydroxypyrene levels after PAH exposure. Carcinogenesis. 21, 669–676. doi: 10.1093/carcin/21.4.669, PMID: 10753202

[B3] Al-Husseini M. J. Kunbaz A. Saad A. M. Santos J. V. Salahia S. Iqbal M. . (2019). Trends in the incidence and mortality of transitional cell carcinoma of the bladder for the last four decades in the USA: a SEER-based analysis. BMC Cancer. 19, 46. doi: 10.1186/s12885-019-5267-3, PMID: 30630456 PMC6327491

[B4] Al-Ishaq R. K. Overy A. J. Büsselberg D. (2020). Phytochemicals and gastrointestinal cancer: cellular mechanisms and effects to change cancer progression. Biomolecules. 10. doi: 10.3390/biom10010105, PMID: 31936288 PMC7022462

[B5] Alouini S. (2024). Risk factors associated with urothelial bladder cancer. Int. J. Environ. Res. Public Health 21:954. doi: 10.3390/ijerph21070954, PMID: 39063530 PMC11277468

[B6] Anderson L. M. Souliotis V. L. Chhabra S. K. Moskal T. J. Harbaugh S. D. Kyrtopoulos S. A. (1996). N-nitrosodimethylamine-derived O(6)-methylguanine in DNA of monkey gastrointestinal and urogenital organs and enhancement by ethanol. Int. J. Cancer. 66, 130–134. doi: 10.1002/(sici)1097-0215(19960328)66:1<130::Aid-ijc22>3.0.Co;2-g, PMID: 8608956

[B7] Antonangeli F. Natalini A. Garassino M. C. Sica A. Santoni A. Di Rosa F. (2020). Regulation of PD-L1 expression by NF-κB in cancer. Front. Immunol. 11. doi: 10.3389/fimmu.2020.584626, PMID: 33324403 PMC7724774

[B8] Armijo A. L. Thongararm P. Fedeles B. I. Yau J. Kay J. E. Corrigan J. J. . (2023). Molecular origins of mutational spectra produced by the environmental carcinogen N-nitrosodimethylamine and S(N)1 chemotherapeutic agents. NAR Cancer 5, zcad015. doi: 10.1093/narcan/zcad015, PMID: 36992846 PMC10041537

[B9] Babjuk M. Burger M. Capoun O. Cohen D. Compérat E. M. Dominguez Escrig J. L. . (2022). European association of urology guidelines on non-muscle-invasive bladder cancer (Ta, T1, and carcinoma in situ). Eur. Urol. 81, 75–94. doi: 10.1016/j.eururo.2021.08.010, PMID: 34511303

[B10] Bacon J. R. Williamson G. Garner R. C. Lappin G. Langouët S. Bao Y. (2003). Sulforaphane and quercetin modulate PhIP-DNA adduct formation in human HepG2 cells and hepatocytes. Carcinogenesis. 24, 1903–1911. doi: 10.1093/carcin/bgg157, PMID: 12949046

[B11] Badawi A. F. Hirvonen A. Bell D. A. Lang N. P. Kadlubar F. F. (1995). Role of aromatic amine acetyltransferases, NAT1 and NAT2, in carcinogen-DNA adduct formation in the human urinary bladder. Cancer Res. 55, 5230–5237., PMID: 7585581

[B12] Bailly C. (2020). Regulation of PD-L1 expression on cancer cells with ROS-modulating drugs. Life Sci. 246, 117403. doi: 10.1016/j.lfs.2020.117403, PMID: 32035131

[B13] Balkwill F. Mantovani A. (2001). Inflammation and cancer: back to Virchow? Lancet 357, 539–545. doi: 10.1016/s0140-6736(00)04046-0, PMID: 11229684

[B14] Bar David Y. Gesundheit B. Urkin J. Kapelushnik J. (2004). Water intake and cancer prevention. J. Clin. Oncol. 22, 383–385. doi: 10.1200/jco.2004.99.245, PMID: 14722055

[B15] Barnes J. L. Zubair M. John K. Poirier M. C. Martin F. L. (2018). Carcinogens and DNA damage. Biochem. Soc. Trans. 46, 1213–1224. doi: 10.1042/bst20180519, PMID: 30287511 PMC6195640

[B16] Bartsch H. Montesano R. (1984). Relevance of nitrosamines to human cancer. Carcinogenesis. 5, 1381–1393. doi: 10.1093/carcin/5.11.1381, PMID: 6386215

[B17] Battaglia A. M. Sacco A. Aversa I. Santamaria G. Palmieri C. Botta C. . (2023). Iron-mediated oxidative stress induces PD-L1 expression via activation of c-Myc in lung adenocarcinoma. Front. Cell Dev. Biol. 11. doi: 10.3389/fcell.2023.1208485, PMID: 37377735 PMC10291098

[B18] Bayat Mokhtari R. Baluch N. Homayouni T. S. Morgatskaya E. Kumar S. Kazemi P. . (2018). The role of Sulforaphane in cancer chemoprevention and health benefits: a mini-review. J. Cell Commun. Signal. 12, 91–101. doi: 10.1007/s12079-017-0401-y, PMID: 28735362 PMC5842175

[B19] Besaratinia A. Tommasi S. (2013). Genotoxicity of tobacco smoke-derived aromatic amines and bladder cancer: current state of knowledge and future research directions. FASEB J. 27, 2090–2100. doi: 10.1096/fj.12-227074, PMID: 23449930

[B20] Beyer A. Biziuk M. (2009). Environmental fate and global distribution of polychlorinated biphenyls. Rev. Environ. Contam Toxicol. 201, 137–158. doi: 10.1007/978-1-4419-0032-6_5, PMID: 19484591

[B21] Bock K. W. Gschaidmeier H. Heel H. Lehmköster T. Münzel P. A. Bock-Hennig B. S. (1999). Functions and transcriptional regulation of PAH-inducible human UDP-glucuronosyltransferases. Drug Metab. Rev. 31, 411–422. doi: 10.1081/dmr-100101927, PMID: 10335444

[B22] Boddupalli S. Mein J. R. Lakkanna S. James D. R. (2012). Induction of phase 2 antioxidant enzymes by broccoli sulforaphane: perspectives in maintaining the antioxidant activity of vitamins a, C, and e. Front. Genet. 3. doi: 10.3389/fgene.2012.00007, PMID: 22303412 PMC3264924

[B23] Boffetta P. Jourenkova N. Gustavsson P. (1997). Cancer risk from occupational and environmental exposure to polycyclic aromatic hydrocarbons. Cancer Causes Control. 8, 444–472. doi: 10.1023/a:1018465507029, PMID: 9498904

[B24] Boots A. W. Haenen G. R. Bast A. (2008). Health effects of quercetin: from antioxidant to nutraceutical. Eur. J. Pharmacol. 585, 325–337. doi: 10.1016/j.ejphar.2008.03.008, PMID: 18417116

[B25] Boström C. E. Gerde P. Hanberg A. Jernström B. Johansson C. Kyrklund T. . (2002). Cancer risk assessment, indicators, and guidelines for polycyclic aromatic hydrocarbons in the ambient air. Environ. Health Perspect. 110 Suppl 3, 451–488. doi: 10.1289/ehp.110-1241197, PMID: 12060843 PMC1241197

[B26] Bruckner J. V. Davis B. D. Blancato J. N. (1989). Metabolism, toxicity, and carcinogenicity of trichloroethylene. Crit. Rev. Toxicol. 20, 31–50. doi: 10.3109/10408448909037475, PMID: 2673291

[B27] Bukavina L. Ginwala R. Eltoukhi M. Sindhani M. Prunty M. Geynisman D. M. . (2024). Role of gut microbiome in neoadjuvant chemotherapy response in urothelial carcinoma: A multi-institutional prospective cohort evaluation. Cancer Res. Commun. 4, 1505–1516. doi: 10.1158/2767-9764.Crc-23-0479, PMID: 38747616 PMC11181990

[B28] Bukowska B. Sicińska P. (2021). Influence of benzo(a)pyrene on different epigenetic processes. Int. J. Mol. Sci. 22:13453. doi: 10.3390/ijms222413453, PMID: 34948252 PMC8707600

[B29] Burger M. Catto J. W. Dalbagni G. Grossman H. B. Herr H. Karakiewicz P. . (2013). Epidemiology and risk factors of urothelial bladder cancer. Eur. Urol. 63, 234–241. doi: 10.1016/j.eururo.2012.07.033, PMID: 22877502

[B30] Bustaffa E. Stoccoro A. Bianchi F. Migliore L. (2014). Genotoxic and epigenetic mechanisms in arsenic carcinogenicity. Arch. Toxicol. 88, 1043–1067. doi: 10.1007/s00204-014-1233-7, PMID: 24691704

[B31] Butt U. A. De Biase D. (2025). The urinary microbiota and the gut-bladder axis in bladder cancer. Int. J. Mol. Sci. 26:10558. doi: 10.3390/ijms262110558, PMID: 41226592 PMC12610541

[B32] Cai H. Shen C. Xu H. Qian H. Pei S. Cai P. . (2023). Seasonal variability, predictive modeling and health risks of N-nitrosamines in drinking water of Shanghai. Sci. Total Environ. 857, 159530. doi: 10.1016/j.scitotenv.2022.159530, PMID: 36270378

[B33] Caliri A. W. Tommasi S. Besaratinia A. (2021). Relationships among smoking, oxidative stress, inflammation, macromolecular damage, and cancer. Mutat. Res. Rev. Mutat. Res. 787, 108365. doi: 10.1016/j.mrrev.2021.108365, PMID: 34083039 PMC8287787

[B34] Callahan C. L. Stewart P. A. Blair A. Purdue M. P. (2019). Extended mortality follow-up of a cohort of dry cleaners. Epidemiology. 30, 285–290. doi: 10.1097/ede.0000000000000951, PMID: 30721169 PMC10402463

[B35] Carpenter D. O. (2006). Polychlorinated biphenyls (PCBs): routes of exposure and effects on human health. Rev. Environ. Health 21, 1–23. doi: 10.1515/reveh.2006.21.1.1, PMID: 16700427

[B36] Chen W. Chen Y. Huang H. Lu Y. Khorram M. S. Zhao W. . (2019). Occurrence of N-Nitrosamines in the Pearl River delta of China: Characterization and evaluation of different sources. Water Res. 164, 114896. doi: 10.1016/j.watres.2019.114896, PMID: 31377526

[B37] Chen Y. Huang H. Chen W. Huang X. Zhang Y. Liang Y. . (2024). Impact of agricultural activities on the occurrence of N-nitrosamines in an aquatic environment. Environ. Sci. Process Impacts. 26, 470–482. doi: 10.1039/d3em00441d, PMID: 38282562

[B38] Chen D. S. Mellman I. (2017). Elements of cancer immunity and the cancer-immune set point. Nature. 541, 321–330. doi: 10.1038/nature21349, PMID: 28102259

[B39] Chen B. Sun L. Zeng G. Shen Z. Wang K. Yin L. . (2022). Gut bacteria alleviate smoking-related NASH by degrading gut nicotine. Nature. 610, 562–568. doi: 10.1038/s41586-022-05299-4, PMID: 36261549 PMC9589931

[B40] Chen W. Teo J. M. N. Yau S. W. Wong M. Y. Lok C. N. Che C. M. . (2022). Chronic type I interferon signaling promotes lipid-peroxidation-driven terminal CD8(+) T cell exhaustion and curtails anti-PD-1 efficacy. Cell Rep. 41, 111647. doi: 10.1016/j.celrep.2022.111647, PMID: 36384131

[B41] Cheng T. Gamage S. M. K. Lu C. T. Aktar S. Gopalan V. Lam A. K. (2022). Polymorphisms in PAH metabolising enzyme CYP1A1 in colorectal cancer and their clinicopathological correlations. Pathol. Res. Pract. 231, 153801. doi: 10.1016/j.prp.2022.153801, PMID: 35180652

[B42] Choi J. W. Kim M. Song G. Kho Y. Choi K. Shin M. Y. . (2023). Toxicokinetic analyses of naphthalene, fluorene, phenanthrene, and pyrene in humans after single oral administration. Sci. Total Environ. 870, 161899. doi: 10.1016/j.scitotenv.2023.161899, PMID: 36716884

[B43] Christoforidou E. P. Riza E. Kales S. N. Hadjistavrou K. Stoltidi M. Kastania A. N. . (2013). Bladder cancer and arsenic through drinking water: a systematic review of epidemiologic evidence. J. Environ. Sci. Health A Tox Hazard Subst Environ. Eng. 48, 1764–1775. doi: 10.1080/10934529.2013.823329, PMID: 24007431

[B44] Clark O. Sarmento T. Eccleston A. Brinkmann J. Picoli R. Daliparthi V. . (2024). Economic impact of bladder cancer in the USA. Pharmacoecon Open 8, 837–845. doi: 10.1007/s41669-024-00512-8, PMID: 39154309 PMC11499469

[B45] Cohen S. M. (1983). Promotion in urinary bladder carcinogenesis. Environ. Health Perspect. 50, 51–59. doi: 10.1289/ehp.835051, PMID: 6873031 PMC1569239

[B46] Collatuzzo G. Hamdani M. Boffetta P. (2024). Risk of bladder, kidney and prostate cancer from occupational exposure to welding fumes: a systematic review and meta-analysis. Int. Arch. Occup. Environ. Health 97, 221–230. doi: 10.1007/s00420-023-02040-0, PMID: 38231405

[B47] Conway L. P. Rendo V. Correia M. S. P. Bergdahl I. A. Sjöblom T. Globisch D. (2020). Unexpected acetylation of endogenous aliphatic amines by arylamine N-acetyltransferase NAT2. Angew Chem. Int. Ed Engl. 59, 14342–14346. doi: 10.1002/anie.202005915, PMID: 32497306 PMC7497018

[B48] Covolo L. Placidi D. Gelatti U. Carta A. Scotto Di Carlo A. Lodetti P. . (2008). Bladder cancer, GSTs, NAT1, NAT2, SULT1A1, XRCC1, XRCC3, XPD genetic polymorphisms and coffee consumption: a case-control study. Eur. J. Epidemiol. 23, 355–362. doi: 10.1007/s10654-008-9238-2, PMID: 18365755

[B49] Cumberbatch M. G. Cox A. Teare D. Catto J. W. (2015). Contemporary occupational carcinogen exposure and bladder cancer: A systematic review and meta-analysis. JAMA Oncol. 1, 1282–1290. doi: 10.1001/jamaoncol.2015.3209, PMID: 26448641

[B50] Cumberbatch M. G. Windsor-Shellard B. Catto J. W. (2017). The contemporary landscape of occupational bladder cancer within the United Kingdom: a meta-analysis of risks over the last 80 years. BJU Int. 119, 100–109. doi: 10.1111/bju.13561, PMID: 27332981

[B51] Das M. (2024). Arsenic contamination and cancer risk in Bangladesh. Lancet Oncol. 25, 538. doi: 10.1016/s1470-2045(24)00156-6, PMID: 38522454

[B52] Das D. N. Ravi N. (2022). Influences of polycyclic aromatic hydrocarbon on the epigenome toxicity and its applicability in human health risk assessment. Environ. Res. 213, 113677. doi: 10.1016/j.envres.2022.113677, PMID: 35714684

[B53] Dhaini H. R. El Hafi B. Khamis A. M. (2018). NAT1 genotypic and phenotypic contribution to urinary bladder cancer risk: a systematic review and meta-analysis. Drug Metab. Rev. 50, 208–219. doi: 10.1080/03602532.2017.1415928, PMID: 29258340

[B54] Di Bona M. Chen Y. Agustinus A. S. Mazzagatti A. Duran M. A. Deyell M. . (2024). Micronuclear collapse from oxidative damage. Science 385, eadj8691. doi: 10.1126/science.adj8691, PMID: 39208110 PMC11610459

[B55] Di Maso M. Bosetti C. Taborelli M. Montella M. Libra M. Zucchetto A. . (2016). Dietary water intake and bladder cancer risk: An Italian case-control study. Cancer Epidemiol. 45, 151–156. doi: 10.1016/j.canep.2016.09.015, PMID: 27821348

[B56] Doolittle D. J. Sherrill J. M. Butterworth B. E. (1983). Influence of intestinal bacteria, sex of the animal, and position of the nitro group on the hepatic genotoxicity of nitrotoluene isomers *in vivo*. Cancer Res. 43, 2836–2842., PMID: 6850595

[B57] Dou K. Xu Q. Han X. (2013). The association between XPC Lys939Gln gene polymorphism and urinary bladder cancer susceptibility: a systematic review and meta-analysis. Diagn. Pathol. 8, 112. doi: 10.1186/1746-1596-8-112, PMID: 23819639 PMC3744163

[B58] Essex M. Rios Rodriguez V. Rademacher J. Proft F. Löber U. Markó L. . (2024). Shared and distinct gut microbiota in spondyloarthritis, acute anterior uveitis, and crohn’s disease. Arthritis Rheumatol. 76, 48–58. doi: 10.1002/art.42658, PMID: 37471465

[B59] Fan Y. Pedersen O. (2021). Gut microbiota in human metabolic health and disease. Nat. Rev. Microbiol. 19, 55–71. doi: 10.1038/s41579-020-0433-9, PMID: 32887946

[B60] Fernández M. I. Brausi M. Clark P. E. Cookson M. S. Grossman H. B. Khochikar M. . (2019). Epidemiology, prevention, screening, diagnosis, and evaluation: update of the ICUD-SIU joint consultation on bladder cancer. World J. Urol. 37, 3–13. doi: 10.1007/s00345-018-2436-y, PMID: 30105454

[B61] Fletcher S. A. Cole A. P. Lu C. Marchese M. Krimphove M. J. Friedlander D. F. . (2020). The impact of underinsurance on bladder cancer diagnosis, survival, and care delivery for individuals under the age of 65 years. Cancer. 126, 496–505. doi: 10.1002/cncr.32562, PMID: 31626340

[B62] Fung T. C. Olson C. A. Hsiao E. Y. (2017). Interactions between the microbiota, immune and nervous systems in health and disease. Nat. Neurosci. 20, 145–155. doi: 10.1038/nn.4476, PMID: 28092661 PMC6960010

[B63] García-Closas M. Malats N. Silverman D. Dosemeci M. Kogevinas M. Hein D. W. . (2005). NAT2 slow acetylation, GSTM1 null genotype, and risk of bladder cancer: results from the Spanish Bladder Cancer Study and meta-analyses. Lancet. 366, 649–659. doi: 10.1016/s0140-6736(05)67137-1, PMID: 16112301 PMC1459966

[B64] García-García C. A. Cruz-Gregorio A. Pedraza-Chaverri J. Montaño L. F. Rendón-Huerta E. P. (2024). NDMA enhances claudin-1 and -6 expression viaCYP2E1/ROS in AGS cells. Toxicol. In Vitro. 102, 105952. doi: 10.1016/j.tiv.2024.105952, PMID: 39395750

[B65] Ghosal A. Iba M. M. (1990). *In vivo* binding of 3,3’-dichlorobenzidine to rat and mouse tissue DNA. Cancer Lett. 53, 197–204. doi: 10.1016/0304-3835(90)90214-i, PMID: 2208080

[B66] Girard H. Butler L. M. Villeneuve L. Millikan R. C. Sinha R. Sandler R. S. . (2008). UGT1A1 and UGT1A9 functional variants, meat intake, and colon cancer, among Caucasians and African-Americans. Mutat. Res. 644, 56–63. doi: 10.1016/j.mrfmmm.2008.07.002, PMID: 18675828 PMC2570038

[B67] Głąbska D. Guzek D. Groele B. Gutkowska K. (2020). Fruit and vegetable intake and mental health in adults: A systematic review. Nutrients. 12:115. doi: 10.3390/nu12010115, PMID: 31906271 PMC7019743

[B68] Golka K. Kopps S. Myslak Z. W. (2004a). Carcinogenicity of azo colorants: influence of solubility and bioavailability. Toxicol. Lett. 151, 203–210. doi: 10.1016/j.toxlet.2003.11.016, PMID: 15177655

[B69] Golka K. Wiese A. Assennato G. Bolt H. M. (2004b). Occupational exposure and urological cancer. World J. Urol. 21, 382–391. doi: 10.1007/s00345-003-0377-5, PMID: 14648102

[B70] Gorski J. C. Wang Z. Haehner-Daniels B. D. Wrighton S. A. Hall S. D. (2000). The effect of hormone replacement therapy on CYP3A activity. Clin. Pharmacol. Ther. 68, 412–417. doi: 10.1067/mcp.2000.110560, PMID: 11061581

[B71] Goyal N. Sridhar J. Do C. Bratton M. Shaik S. Jiang Q. . (2021). Identification of CYP 2A6 inhibitors in an effort to mitigate the harmful effects of the phytochemical nicotine. J. Cancer Metastasis Treat 7:18. doi: 10.20517/2394-4722.2020.143, PMID: 34722929 PMC8555909

[B72] Grimm F. A. Hu D. Kania-Korwel I. Lehmler H. J. Ludewig G. Hornbuckle K. C. . (2015). Metabolism and metabolites of polychlorinated biphenyls. Crit. Rev. Toxicol. 45, 245–272. doi: 10.3109/10408444.2014.999365, PMID: 25629923 PMC4383295

[B73] Guyton K. Z. Hogan K. A. Scott C. S. Cooper G. S. Bale A. S. Kopylev L. . (2014). Human health effects of tetrachloroethylene: key findings and scientific issues. Environ. Health Perspect. 122, 325–334. doi: 10.1289/ehp.1307359, PMID: 24531164 PMC3984230

[B74] Habil M. R. Salazar-González R. A. Doll M. A. Hein D. W. (2022). Differences in β-naphthylamine metabolism and toxicity in Chinese hamster ovary cell lines transfected with human CYP1A2 and NAT2*4, NAT2*5B or NAT2*7B N-acetyltransferase 2 haplotypes. Arch. Toxicol. 96, 2999–3012. doi: 10.1007/s00204-022-03367-2, PMID: 36040704 PMC10187863

[B75] Haiser H. J. Turnbaugh P. J. (2012). Is it time for a metagenomic basis of therapeutics? Science 336, 1253–1255. doi: 10.1126/science.1224396, PMID: 22674325

[B76] Haiser H. J. Turnbaugh P. J. (2013). Developing a metagenomic view of xenobiotic metabolism. Pharmacol. Res. 69, 21–31. doi: 10.1016/j.phrs.2012.07.009, PMID: 22902524 PMC3526672

[B77] Hamidi H. Ivaska J. (2018). Every step of the way: integrins in cancer progression and metastasis. Nat. Rev. Cancer. 18, 533–548. doi: 10.1038/s41568-018-0038-z, PMID: 30002479 PMC6629548

[B78] Han S. Van Treuren W. Fischer C. R. Merrill B. D. DeFelice B. C. Sanchez J. M. . (2021). A metabolomics pipeline for the mechanistic interrogation of the gut microbiome. Nature. 595, 415–420. doi: 10.1038/s41586-021-03707-9, PMID: 34262212 PMC8939302

[B79] Han H. Wolff E. M. Liang G. (2012). Epigenetic alterations in bladder cancer and their potential clinical implications. Adv. Urol. 2012, 546917. doi: 10.1155/2012/546917, PMID: 22829811 PMC3397159

[B80] Hardt L. Mahamat-Saleh Y. Aune D. Schlesinger S. (2022). Plant-based diets and cancer prognosis: a review of recent research. Curr. Nutr. Rep. 11, 695–716. doi: 10.1007/s13668-022-00440-1, PMID: 36138327 PMC9750928

[B81] Haschek-Hock W. M. Rousseaux C. G. Wallig M. A. Bolon B. (2022). NEW: haschek and rousseaux’s handbook of toxicologic pathology, volume 1: principles and practice of toxicologic pathology, 4th edition - 2021. Int. J. Toxicol. 41, 253–254. doi: 10.1177/10915818221099126, PMID: 35514322

[B82] Hathway D. E. (2000). Toxic action/toxicity. Biol. Rev. Camb Philos. Soc 75, 95–127. doi: 10.1017/s0006323199005447, PMID: 10740894

[B83] He C. Li B. Huang L. Teng C. Bao Y. Ren M. . (2020). Gut microbial composition changes in bladder cancer patients: A case-control study in Harbin, China. Asia Pac J. Clin. Nutr. 29, 395–403. doi: 10.6133/apjcn.202007_29(2).0022, PMID: 32674247

[B84] He J. Shi T. Y. Zhu M. L. Wang M. Y. Li Q. X. Wei Q. Y. (2013). Associations of Lys939Gln and Ala499Val polymorphisms of the XPC gene with cancer susceptibility: a meta-analysis. Int. J. Cancer. 133, 1765–1775. doi: 10.1002/ijc.28089, PMID: 23400628

[B85] Hein D. W. (2002). Molecular genetics and function of NAT1 and NAT2: role in aromatic amine metabolism and carcinogenesis. Mutat. Res. 506-507, 65–77. doi: 10.1016/s0027-5107(02)00153-7, PMID: 12351146

[B86] Hein D. W. Doll M. A. Fretland A. J. Leff M. A. Webb S. J. Xiao G. H. . (2000). Molecular genetics and epidemiology of the NAT1 and NAT2 acetylation polymorphisms. Cancer Epidemiol. Biomarkers Prev. 9, 29–42. 10667461

[B87] Hicks R. M. Ismail M. M. Walters C. L. Beecham P. T. Rabie M. F. El Alamy M. A. (1982). Association of bacteriuria and urinary nitrosamine formation with Schistosoma haematobium infection in the Qalyub area of Egypt. Trans. R Soc. Trop. Med. Hyg. 76, 519–527. doi: 10.1016/0035-9203(82)90153-5, PMID: 6926771

[B88] Hour T. C. Huang C. Y. Lin C. C. Chen J. Guan J. Y. Lee J. M. . (2004). Characterization of molecular events in a series of bladder urothelial carcinoma cell lines with progressive resistance to arsenic trioxide. Anticancer Drugs 15, 779–785. doi: 10.1097/00001813-200409000-00007, PMID: 15494640

[B89] Hughes D. A. Bacigalupe R. Wang J. Rühlemann M. C. Tito R. Y. Falony G. . (2020). Genome-wide associations of human gut microbiome variation and implications for causal inference analyses. Nat. Microbiol. 5, 1079–1087. doi: 10.1038/s41564-020-0743-8, PMID: 32572223 PMC7610462

[B90] Hung R. J. Boffetta P. Brennan P. Malaveille C. Hautefeuille A. Donato F. . (2004). NAT, SULT1A1, CYP1B1 genetic polymorphisms, interactions with environmental exposures and bladder cancer risk in a high-risk population. Int. J. Cancer. 110, 598–604. doi: 10.1002/ijc.20157, PMID: 15122594

[B91] Imaoka S. Yoneda Y. Matsuda T. Degawa M. Fukushima S. Funae Y. (1997). Mutagenic activation of urinary bladder carcinogens by CYP4B1 and the presence of CYP4B1 in bladder mucosa. Biochem. Pharmacol. 54, 677–683. doi: 10.1016/s0006-2952(97)00216-5, PMID: 9310344

[B92] Isali I. Helstrom E. K. Uzzo N. Lakshmanan A. Nandwana D. Valentine H. . (2024). Current trends and challenges of microbiome research in bladder cancer. Curr. Oncol. Rep. 26, 292–298. doi: 10.1007/s11912-024-01508-7, PMID: 38376627 PMC10920447

[B93] Izumi K. Zheng Y. Hsu J. W. Chang C. Miyamoto H. (2013). Androgen receptor signals regulate UDP-glucuronosyltransferases in the urinary bladder: a potential mechanism of androgen-induced bladder carcinogenesis. Mol. Carcinog. 52, 94–102. doi: 10.1002/mc.21833, PMID: 22086872

[B94] Jin F. Thaiparambil J. Donepudi S. R. Vantaku V. Piyarathna D. W. B. Maity S. . (2017). Tobacco-specific carcinogens induce hypermethylation, DNA adducts, and DNA damage in bladder cancer. Cancer Prev. Res. (Phila). 10, 588–597. doi: 10.1158/1940-6207.Capr-17-0198, PMID: 28851690 PMC5626664

[B95] Joyce D. D. Sharma V. Williams S. B. (2023). Cost-effectiveness and economic impact of bladder cancer management: an updated review of the literature. Pharmacoeconomics. 41, 751–769. doi: 10.1007/s40273-023-01273-8, PMID: 37088844

[B96] Kakizoe T. Wang T. T. Eng V. W. Furrer R. Dion P. Bruce W. R. (1979). Volatile N-nitrosamines in the urine of normal donors and of bladder cancer patients. Cancer Res. 39, 829–832., PMID: 427771

[B97] Kamada N. Chen G. Y. Inohara N. Núñez G. (2013). Control of pathogens and pathobionts by the gut microbiota. Nat. Immunol. 14, 685–690. doi: 10.1038/ni.2608, PMID: 23778796 PMC4083503

[B98] Karin M. Greten F. R. (2005). NF-kappaB: linking inflammation and immunity to cancer development and progression. Nat. Rev. Immunol. 5, 749–759. doi: 10.1038/nri1703, PMID: 16175180

[B99] Kastrinou Lampou V. Poller B. Huth F. Fischer A. Kullak-Ublick G. A. Arand M. . (2023). Novel insights into bile acid detoxification via CYP, UGT and SULT enzymes. Toxicol. In Vitro. 87, 105533. doi: 10.1016/j.tiv.2022.105533, PMID: 36473578

[B100] Keimling M. Behrens G. Schmid D. Jochem C. Leitzmann M. F. (2014). The association between physical activity and bladder cancer: systematic review and meta-analysis. Br. J. Cancer. 110, 1862–1870. doi: 10.1038/bjc.2014.77, PMID: 24594995 PMC3974090

[B101] Kelly D. M. Jones T. H. (2013). Testosterone: a metabolic hormone in health and disease. J. Endocrinol. 217, R25–R45. doi: 10.1530/joe-12-0455, PMID: 23378050

[B102] Khan S. U. Fatima K. Malik F. Kalkavan H. Wani A. (2023). Cancer metastasis: Molecular mechanisms and clinical perspectives. Pharmacol. Ther. 250, 108522. doi: 10.1016/j.pharmthera.2023.108522, PMID: 37661054

[B103] Kim K. H. Jahan S. A. Kabir E. Brown R. J. (2013). A review of airborne polycyclic aromatic hydrocarbons (PAHs) and their human health effects. Environ. Int. 60, 71–80. doi: 10.1016/j.envint.2013.07.019, PMID: 24013021

[B104] Kim K. S. Lee Y. M. Kim S. G. Lee I. K. Lee H. J. Kim J. H. . (2014). Associations of organochlorine pesticides and polychlorinated biphenyls in visceral vs. subcutaneous adipose tissue with type 2 diabetes and insulin resistance. Chemosphere. 94, 151–157. doi: 10.1016/j.chemosphere.2013.09.066, PMID: 24161582

[B105] Kiriluk K. J. Prasad S. M. Patel A. R. Steinberg G. D. Smith N. D. (2012). Bladder cancer risk from occupational and environmental exposures. Urol Oncol. 30, 199–211. doi: 10.1016/j.urolonc.2011.10.010, PMID: 22385990

[B106] Klein E. A. Thompson I. M. Jr. Tangen C. M. Crowley J. J. Lucia M. S. Goodman P. J. . (2011). Vitamin E and the risk of prostate cancer: the Selenium and Vitamin E Cancer Prevention Trial (SELECT). Jama. 306, 1549–1556. doi: 10.1001/jama.2011.1437, PMID: 21990298 PMC4169010

[B107] Kogevinas M. t Mannetje A. Cordier S. Ranft U. González C. A. Vineis P. . (2003). Occupation and bladder cancer among men in Western Europe. Cancer Causes Control. 14, 907–914. doi: 10.1023/b:caco.0000007962.19066.9c, PMID: 14750529

[B108] Kolodziejczyk A. A. Zheng D. Elinav E. (2019). Diet-microbiota interactions and personalized nutrition. Nat. Rev. Microbiol. 17, 742–753. doi: 10.1038/s41579-019-0256-8, PMID: 31541197

[B109] Kurokawa M. MacLeod M. C. (1985). Differences in the binding of stereoisomeric benzo(a)pyrene-7,8-diol-9,10-epoxides to histones in rat liver nuclei. Cancer Res. 45, 5741–5745., PMID: 3931908

[B110] Kurosawa A. Saito S. Sakurai M. Shinozuka M. Someya Y. Adachi N. (2023). Arsenic affects homologous recombination and single-strand annealing but not end-joining pathways during DNA double-strand break repair. FEBS J. 290, 5313–5321. doi: 10.1111/febs.16922, PMID: 37530740

[B111] Kustrimovic N. Bilato G. Mortara L. Baci D. (2024). The urinary microbiome in health and disease: relevance for bladder cancer. Int. J. Mol. Sci. 25:1732. doi: 10.3390/ijms25031732, PMID: 38339010 PMC10855347

[B112] Lambert J. D. Elias R. J. (2010). The antioxidant and pro-oxidant activities of green tea polyphenols: a role in cancer prevention. Arch. Biochem. Biophys. 501, 65–72. doi: 10.1016/j.abb.2010.06.013, PMID: 20558130 PMC2946098

[B113] Lang Y. Li P. He R. Zhu B. Wang G. Li J. (2025). Dynamic changes of urinary microbiota in patients with bladder cancer after surgery and its clinical significance. Front. Immunol. 16. doi: 10.3389/fimmu.2025.1638628, PMID: 40959077 PMC12434072

[B114] Lash L. H. Chiu W. A. Guyton K. Z. Rusyn I. (2014). Trichloroethylene biotransformation and its role in mutagenicity, carcinogenicity and target organ toxicity. Mutat. Res. Rev. Mutat. Res. 762, 22–36. doi: 10.1016/j.mrrev.2014.04.003, PMID: 25484616 PMC4254735

[B115] Lash L. H. Fisher J. W. Lipscomb J. C. Parker J. C. (2000). Metabolism of trichloroethylene. Environ. Health Perspect. 108 Suppl 2, 177–200. doi: 10.1289/ehp.00108s2177, PMID: 10807551 PMC1637769

[B116] Lash L. H. Parker J. C. (2001). Hepatic and renal toxicities associated with perchloroethylene. Pharmacol. Rev. 53, 177–208. doi: 10.1016/S0031-6997(24)01491-1 11356983

[B117] Leal J. Luengo-Fernandez R. Sullivan R. Witjes J. A. (2016). Economic burden of bladder cancer across the european union. Eur. Urol. 69, 438–447. doi: 10.1016/j.eururo.2015.10.024, PMID: 26508308

[B118] Lee S. Y. Jeong E. K. Ju M. K. Jeon H. M. Kim M. Y. Kim C. H. . (2017). Induction of metastasis, cancer stem cell phenotype, and oncogenic metabolism in cancer cells by ionizing radiation. Mol. Cancer. 16, 10. doi: 10.1186/s12943-016-0577-4, PMID: 28137309 PMC5282724

[B119] Lee L. J. Kwon C. S. Forsythe A. Mamolo C. M. Masters E. T. Jacobs I. A. (2020). Humanistic and economic burden of non-muscle invasive bladder cancer: results of two systematic literature reviews. Clinicoecon Outcomes Res. 12, 693–709. doi: 10.2147/ceor.S274951, PMID: 33262624 PMC7695604

[B120] Leon M. E. Peruga A. McNeill A. Kralikova E. Guha N. Minozzi S. . (2015). European Code against Cancer, 4th Edition: Tobacco and cancer. Cancer Epidemiol. 39 Suppl 1, S20–S33. doi: 10.1016/j.canep.2015.06.001, PMID: 26272517

[B121] Li J. Jia H. Cai X. Zhong H. Feng Q. Sunagawa S. . (2014). An integrated catalog of reference genes in the human gut microbiome. Nat. Biotechnol. 32, 834–841. doi: 10.1038/nbt.2942, PMID: 24997786

[B122] Li D. Wang L. E. Chang P. El-Naggar A. K. Sturgis E. M. Wei Q. (2007). *In vitro* benzo[a]pyrene diol epoxide-induced DNA adducts and risk of squamous cell carcinoma of head and neck. Cancer Res. 67, 5628–5634. doi: 10.1158/0008-5472.Can-07-0983, PMID: 17575128

[B123] Li H. Yuan Y. Zhang L. Xu C. Xu H. Chen Z. (2024). Reprogramming macrophage polarization, depleting ROS by astaxanthin and thioketal-containing polymers delivering rapamycin for osteoarthritis treatment. Adv. Sci. (Weinh). 11, e2305363. doi: 10.1002/advs.202305363, PMID: 38093659 PMC10916582

[B124] Li W. Zeng Q. Wang B. Lv C. He H. Yang X. . (2024). Oxidative stress promotes oral carcinogenesis via Thbs1-mediated M1-like tumor-associated macrophages polarization. Redox Biol. 76, 103335. doi: 10.1016/j.redox.2024.103335, PMID: 39255693 PMC11414564

[B125] Liu J. Wei Y. Jia W. Can C. Wang R. Yang X. . (2022). Chenodeoxycholic acid suppresses AML progression through promoting lipid peroxidation via ROS/p38 MAPK/DGAT1 pathway and inhibiting M2 macrophage polarization. Redox Biol. 56, 102452. doi: 10.1016/j.redox.2022.102452, PMID: 36084349 PMC9465103

[B126] Lloyd L. (2024). Gut microbiota influence bladder tumour development. Nat. Rev. Urol. 21, 576. doi: 10.1038/s41585-024-00947-y, PMID: 39284938

[B127] Lobo N. Afferi L. Moschini M. Mostafid H. Porten S. Psutka S. P. . (2022). Epidemiology, screening, and prevention of bladder cancer. Eur. Urol Oncol. 5, 628–639. doi: 10.1016/j.euo.2022.10.003, PMID: 36333236

[B128] Lock E. A. Reed C. J. (2006). Trichloroethylene: mechanisms of renal toxicity and renal cancer and relevance to risk assessment. Toxicol. Sci. 91, 313–331. doi: 10.1093/toxsci/kfj107, PMID: 16421178

[B129] Lu Y. Chen Z. F. Chen Y. J. Xu Y. Z. Chen Y. Dai X. . (2021). Distribution and risk assessment of hexachlorobutadiene, pentachloroanisole, and chlorobenzenes in sediment and wild fish from a region affected by industrial and agricultural activities in South China. J. Hazard Mater. 417, 126002. doi: 10.1016/j.jhazmat.2021.126002, PMID: 33992918

[B130] Macholz R. Kujawa M. Schulze J. Lewerenz H. J. Schnaak W. (1985). The metabolism of some xenobiotics in germ-free and conventional rats. Arch. Toxicol. Suppl. 8, 373–376. doi: 10.1007/978-3-642-69928-3_77, PMID: 2420308

[B131] Madrid F. Rubio-Bellido M. Morillo E. (2020). Extraction of nonylphenol, pyrene and phenanthrene from sewage sludge and composted biosolids by cyclodextrins and rhamnolipids. Sci. Total Environ. 715, 136986. doi: 10.1016/j.scitotenv.2020.136986, PMID: 32023519

[B132] Maj T. Wang W. Crespo J. Zhang H. Wang W. Wei S. . (2017). Oxidative stress controls regulatory T cell apoptosis and suppressor activity and PD-L1-blockade resistance in tumor. Nat. Immunol. 18, 1332–1341. doi: 10.1038/ni.3868, PMID: 29083399 PMC5770150

[B133] Mani S. (2024). Gut microbiome and bladder cancer: A new link through nitrosamine metabolism. Cell Host Microbe 32, 1637–1639. doi: 10.1016/j.chom.2024.09.003, PMID: 39389023

[B134] Mann D. B. Springer D. L. Smerdon M. J. (1997). DNA damage can alter the stability of nucleosomes: effects are dependent on damage type. Proc. Natl. Acad. Sci. U S A. 94, 2215–2220. doi: 10.1073/pnas.94.6.2215, PMID: 9122174 PMC20067

[B135] Marsit C. J. Karagas M. R. Schned A. Kelsey K. T. (2006). Carcinogen exposure and epigenetic silencing in bladder cancer. Ann. N Y Acad. Sci. 1076, 810–821. doi: 10.1196/annals.1371.031, PMID: 17119258

[B136] Mastrangelo G. Fadda E. Marzia V. (1996). Polycyclic aromatic hydrocarbons and cancer in man. Environ. Health Perspect. 104, 1166–1170. doi: 10.1289/ehp.961041166, PMID: 8959405 PMC1469515

[B137] McCarty K. M. Santella R. M. Steck S. E. Cleveland R. J. Ahn J. Ambrosone C. B. . (2009). PAH-DNA adducts, cigarette smoking, GST polymorphisms, and breast cancer risk. Environ. Health Perspect. 117, 552–558. doi: 10.1289/ehp.0800119, PMID: 19440493 PMC2679598

[B138] Michaud D. S. Spiegelman D. Clinton S. K. Rimm E. B. Curhan G. C. Willett W. C. . (1999). Fluid intake and the risk of bladder cancer in men. N Engl. J. Med. 340, 1390–1397. doi: 10.1056/nejm199905063401803, PMID: 10228189

[B139] Minchin R. F. Butcher N. J. (2015). The role of lysine(100) in the binding of acetylcoenzyme A to human arylamine N-acetyltransferase 1: implications for other acetyltransferases. Biochem. Pharmacol. 94, 195–202. doi: 10.1016/j.bcp.2015.01.015, PMID: 25660616

[B140] Mingdong W. Xiang G. Yongjun Q. Mingshuai W. Hao P. (2023). Causal associations between gut microbiota and urological tumors: a two-sample mendelian randomization study. BMC Cancer. 23, 854. doi: 10.1186/s12885-023-11383-3, PMID: 37697271 PMC10496293

[B141] Mirvish S. S. (1995). Role of N-nitroso compounds (NOC) and N-nitrosation in etiology of gastric, esophageal, nasopharyngeal and bladder cancer and contribution to cancer of known exposures to NOC. Cancer Lett. 93, 17–48. doi: 10.1016/0304-3835(95)03786-v, PMID: 7600541

[B142] Mittal M. Siddiqui M. R. Tran K. Reddy S. P. Malik A. B. (2014). Reactive oxygen species in inflammation and tissue injury. Antioxid Redox Signal. 20, 1126–1167. doi: 10.1089/ars.2012.5149, PMID: 23991888 PMC3929010

[B143] Miyake M. Oda Y. Owari T. Iida K. Ohnishi S. Fujii T. . (2023). Probiotics enhances anti-tumor immune response induced by gemcitabine plus cisplatin chemotherapy for urothelial cancer. Cancer Sci. 114, 1118–1130. doi: 10.1111/cas.15666, PMID: 36398663 PMC9986082

[B144] Möller L. (1994). *In vivo* metabolism and genotoxic effects of nitrated polycyclic aromatic hydrocarbons. Environ. Health Perspect. 102 Suppl 4, 139–146. doi: 10.1289/ehp.102-1566915, PMID: 7821288 PMC1566915

[B145] Möller L. Corrie M. Midtvedt T. Rafter J. Gustafsson J. A. (1988). The role of the intestinal microflora in the formation of mutagenic metabolites from the carcinogenic air pollutant 2-nitrofluorene. Carcinogenesis. 9, 823–830. doi: 10.1093/carcin/9.5.823, PMID: 3365841

[B146] Montano L. Pironti C. Pinto G. Ricciardi M. Buono A. Brogna C. . (2022). Polychlorinated biphenyls (PCBs) in the environment: occupational and exposure events, effects on human health and fertility. Toxics 10:365. doi: 10.3390/toxics10070365, PMID: 35878270 PMC9323099

[B147] Moore L. E. Boffetta P. Karami S. Brennan P. Stewart P. S. Hung R. . (2010). Occupational trichloroethylene exposure and renal carcinoma risk: evidence of genetic susceptibility by reductive metabolism gene variants. Cancer Res. 70, 6527–6536. doi: 10.1158/0008-5472.Can-09-4167, PMID: 20663906 PMC2922418

[B148] Moore B. P. Hicks R. M. Knowles M. A. Redgrave S. (1982). Metabolism and binding of benzo(a)pyrene and 2-acetylaminofluorene by short-term organ cultures of human and rat bladder. Cancer Res. 42, 642–648., PMID: 6275985

[B149] Moorthy B. Chu C. Carlin D. J. (2015). Polycyclic aromatic hydrocarbons: from metabolism to lung cancer. Toxicol. Sci. 145, 5–15. doi: 10.1093/toxsci/kfv040, PMID: 25911656 PMC4408964

[B150] Morrison D. J. Preston T. (2016). Formation of short chain fatty acids by the gut microbiota and their impact on human metabolism. Gut Microbes 7, 189–200. doi: 10.1080/19490976.2015.1134082, PMID: 26963409 PMC4939913

[B151] Mossanen M. Gore J. L. (2014). The burden of bladder cancer care: direct and indirect costs. Curr. Opin. Urol. 24, 487–491. doi: 10.1097/mou.0000000000000078, PMID: 24887047

[B152] Motohashi N. Nagashima H. Molnár J. (1999). Trichloroethylene. III. Prediction of carcinogenicity of investigated compounds including trichloroethylene. In Vivo. 13, 221–224., PMID: 10459495

[B153] Muenyi C. S. Ljungman M. States J. C. (2015). Arsenic disruption of DNA damage responses-potential role in carcinogenesis and chemotherapy. Biomolecules. 5, 2184–2193. doi: 10.3390/biom5042184, PMID: 26404387 PMC4693233

[B154] Nakamura I. Hosokawa K. Tamura H. Miura T. (1977). Reduced mercury excretion with feces in germfree mice after oral administration of methyl mercury chloride. Bull. Environ. Contam Toxicol. 17, 528–533. doi: 10.1007/bf01685974, PMID: 861407

[B155] Navas L. E. Carnero A. (2021). NAD(+) metabolism, stemness, the immune response, and cancer. Signal Transduct Target Ther. 6, 2. doi: 10.1038/s41392-020-00354-w, PMID: 33384409 PMC7775471

[B156] Neumann H. G. (2010). Aromatic amines: mechanisms of carcinogenesis and implications for risk assessment. Front. Biosci. (Landmark Ed). 15, 1119–1130. doi: 10.2741/3665, PMID: 20515745

[B157] Okaru A. O. Lachenmeier D. W. (2021). Margin of exposure analyses and overall toxic effects of alcohol with special consideration of carcinogenicity. Nutrients. 13:3785. doi: 10.3390/nu13113785, PMID: 34836041 PMC8619253

[B158] Ozturk M. Metin M. Altay V. Bhat R. A. Ejaz M. Gul A. . (2022). Arsenic and human health: genotoxicity, epigenomic effects, and cancer signaling. Biol. Trace Elem Res. 200, 988–1001. doi: 10.1007/s12011-021-02719-w, PMID: 33864199

[B159] Pallares-Mendez R. Brassetti A. Bove A. M. Simone G. (2024). Insights into the interplay between the urinary microbiome and bladder cancer: A comprehensive review. J. Clin. Med. 13:4927. doi: 10.3390/jcm13164927, PMID: 39201069 PMC11355659

[B160] Pavanello S. Mastrangelo G. Placidi D. Campagna M. Pulliero A. Carta A. . (2010). CYP1A2 polymorphisms, occupational and environmental exposures and risk of bladder cancer. Eur. J. Epidemiol. 25, 491–500. doi: 10.1007/s10654-010-9479-8, PMID: 20559687

[B161] Pegg A. E. (2011). Multifaceted roles of alkyltransferase and related proteins in DNA repair, DNA damage, resistance to chemotherapy, and research tools. Chem. Res. Toxicol. 24, 618–639. doi: 10.1021/tx200031q, PMID: 21466232 PMC3095683

[B162] Plöttner S. Bastian L. A. Käfferlein H. U. Brüning T. (2016). Effects of benzo[a]pyrene, aromatic amines, and a combination of both on CYP1A1 activities in RT-4 human bladder papilloma cells. J. Toxicol. Environ. Health A 79, 1106–1117. doi: 10.1080/15287394.2016.1219598, PMID: 27924717

[B163] Poirier M. C. (1997). DNA adducts as exposure biomarkers and indicators of cancer risk. Environ. Health Perspect. 105 Suppl 4, 907–912. doi: 10.1289/ehp.97105s4907, PMID: 9255579 PMC1470061

[B164] Prasad S. Gupta S. C. Tyagi A. K. (2017). Reactive oxygen species (ROS) and cancer: Role of antioxidative nutraceuticals. Cancer Lett. 387, 95–105. doi: 10.1016/j.canlet.2016.03.042, PMID: 27037062

[B165] Pritykin Y. van der Veeken J. Pine A. R. Zhong Y. Sahin M. Mazutis L. . (2021). A unified atlas of CD8 T cell dysfunctional states in cancer and infection. Mol. Cell. 81, 2477–2493.e2410. doi: 10.1016/j.molcel.2021.03.045, PMID: 33891860 PMC8454502

[B166] Qin J. Li R. Raes J. Arumugam M. Burgdorf K. S. Manichanh C. . (2010). A human gut microbial gene catalogue established by metagenomic sequencing. Nature. 464, 59–65. doi: 10.1038/nature08821, PMID: 20203603 PMC3779803

[B167] Rafii F. Franklin W. Cerniglia C. E. (1990). Azoreductase activity of anaerobic bacteria isolated from human intestinal microflora. Appl. Environ. Microbiol. 56, 2146–2151. doi: 10.1128/aem.56.7.2146-2151.1990, PMID: 2202258 PMC184574

[B168] Rafil F. Franklin W. Heflich R. H. Cerniglia C. E. (1991). Reduction of nitroaromatic compounds by anaerobic bacteria isolated from the human gastrointestinal tract. Appl. Environ. Microbiol. 57, 962–968. doi: 10.1128/aem.57.4.962-968.1991, PMID: 2059053 PMC182830

[B169] Rafnar T. Sulem P. Stacey S. N. Geller F. Gudmundsson J. Sigurdsson A. . (2009). Sequence variants at the TERT-CLPTM1L locus associate with many cancer types. Nat. Genet. 41, 221–227. doi: 10.1038/ng.296, PMID: 19151717 PMC4525478

[B170] Reed O. Jubber I. Griffin J. Noon A. P. Goodwin L. Hussain S. . (2020). Occupational bladder cancer: A cross section survey of previous employments, tasks and exposures matched to cancer phenotypes. PloS One 15, e0239338. doi: 10.1371/journal.pone.0239338, PMID: 33085669 PMC7577448

[B171] Renwick A. G. Drasar B. S. (1976). Environmental carcinogens and large bowel cancer. Nature. 263, 234–235. doi: 10.1038/263234a0, PMID: 958475

[B172] Reuter S. Gupta S. C. Chaturvedi M. M. Aggarwal B. B. (2010). Oxidative stress, inflammation, and cancer: how are they linked? Free Radic. Biol. Med. 49, 1603–1616. doi: 10.1016/j.freeradbiomed.2010.09.006, PMID: 20840865 PMC2990475

[B173] Roje B. Zhang B. Mastrorilli E. Kovačić A. Sušak L. Ljubenkov I. . (2024). Gut microbiota carcinogen metabolism causes distal tissue tumours. Nature 632, 1137–1144. doi: 10.1038/s41586-024-07754-w, PMID: 39085612 PMC11358042

[B174] Roldán M. D. Pérez-Reinado E. Castillo F. Moreno-Vivián C. (2008). Reduction of polynitroaromatic compounds: the bacterial nitroreductases. FEMS Microbiol. Rev. 32, 474–500. doi: 10.1111/j.1574-6976.2008.00107.x, PMID: 18355273

[B175] Roos P. H. Bolt H. M. (2005). Cytochrome P450 interactions in human cancers: new aspects considering CYP1B1. Expert Opin. Drug Metab. Toxicol. 1, 187–202. doi: 10.1517/17425255.1.2.187, PMID: 16922636

[B176] Ros M. M. Bas Bueno-de-Mesquita H. B. Büchner F. L. Aben K. K. Kampman E. Egevad L. . (2011). Fluid intake and the risk of urothelial cell carcinomas in the European Prospective Investigation into Cancer and Nutrition (EPIC). Int. J. Cancer. 128, 2695–2708. doi: 10.1002/ijc.25592, PMID: 20715171

[B177] Rothschild D. Weissbrod O. Barkan E. Kurilshikov A. Korem T. Zeevi D. . (2018). Environment dominates over host genetics in shaping human gut microbiota. Nature. 555, 210–215. doi: 10.1038/nature25973, PMID: 29489753

[B178] Rouissi K. Bahria I. B. Bougatef K. Marrakchi R. Stambouli N. Hamdi K. . (2011). The effect of tobacco, XPC, ERCC2 and ERCC5 genetic variants in bladder cancer development. BMC Cancer. 11, 101. doi: 10.1186/1471-2407-11-101, PMID: 21426550 PMC3068124

[B179] Rowland I. R. Davies M. J. (1981). *In vitro* metabolism of inorganic arsenic by the gastro-intestinal microflora of the rat. J. Appl. Toxicol. 1, 278–283. doi: 10.1002/jat.2550010508, PMID: 7185888

[B180] Rushton L. Bagga S. Bevan R. Brown T. P. Cherrie J. W. Holmes P. . (2010). Occupation and cancer in britain. Br. J. Cancer. 102, 1428–1437. doi: 10.1038/sj.bjc.6605637, PMID: 20424618 PMC2865752

[B181] Rusyn I. Chiu W. A. Lash L. H. Kromhout H. Hansen J. Guyton K. Z. (2014). Trichloroethylene: Mechanistic, epidemiologic and other supporting evidence of carcinogenic hazard. Pharmacol. Ther. 141, 55–68. doi: 10.1016/j.pharmthera.2013.08.004, PMID: 23973663 PMC3867557

[B182] Safiri S. Kolahi A. A. Naghavi M. (2021). Global, regional and national burden of bladder cancer and its attributable risk factors in 204 countries and territories, 1990-2019: a systematic analysis for the Global Burden of Disease study 2019. BMJ Glob Health 6:e004128. doi: 10.1136/bmjgh-2020-004128, PMID: 34844997 PMC8634015

[B183] Salech F. Ponce D. P. Paula-Lima A. C. SanMartin C. D. Behrens M. I. (2020). Nicotinamide, a poly [ADP-ribose] polymerase 1 (PARP-1) inhibitor, as an adjunctive therapy for the treatment of alzheimer’s disease. Front. Aging Neurosci. 12. doi: 10.3389/fnagi.2020.00255, PMID: 32903806 PMC7438969

[B184] Salinas-Sánchez A. S. Sánchez-Sánchez F. Donate-Moreno M. J. Rubio-del-Campo A. Gimenez-Bachs J. M. Lorenzo-Romero J. G. . (2011). Polymorphic deletions of the GSTT1 and GSTM1 genes and susceptibility to bladder cancer. BJU Int. 107, 1825–1832. doi: 10.1111/j.1464-410X.2010.09683.x, PMID: 20942828

[B185] Scharping N. E. Rivadeneira D. B. Menk A. V. Vignali P. D. A. Ford B. R. Rittenhouse N. L. . (2021). Mitochondrial stress induced by continuous stimulation under hypoxia rapidly drives T cell exhaustion. Nat. Immunol. 22, 205–215. doi: 10.1038/s41590-020-00834-9, PMID: 33398183 PMC7971090

[B186] Scheline R. R. (1973). Metabolism of foreign compounds by gastrointestinal microorganisms. Pharmacol. Rev. 25, 451–523. doi: 10.1016/S0031-6997(25)06622-0, PMID: 4587548

[B187] Schrenk D. Bignami M. Bodin L. Chipman J. K. Del Mazo J. Hogstrand C. . (2023). Risk assessment of N-nitrosamines in food. Efsa J. 21, e07884. doi: 10.2903/j.efsa.2023.7884, PMID: 36999063 PMC10043641

[B188] Sciannameo V. Carta A. d’Errico A. Giraudo M. T. Fasanelli F. Arici C. . (2019). New insights on occupational exposure and bladder cancer risk: a pooled analysis of two Italian case-control studies. Int. Arch. Occup. Environ. Health 92, 347–359. doi: 10.1007/s00420-018-1388-2, PMID: 30506367

[B189] Sculley T. B. Zytkovicz T. H. (1983). Binding of benzo(a)pyrene and (+/-)-7 beta,8 alpha-dihydroxy-9 alpha, 10 alpha-epoxy-7,8,9, 10-tetrahydrobenzo(a)pyrene to histones. Cancer Res. 43, 1688–1695., PMID: 6299528

[B190] Sender R. Fuchs S. Milo R. (2016). Revised estimates for the number of human and bacteria cells in the body. PloS Biol. 14, e1002533. doi: 10.1371/journal.pbio.1002533, PMID: 27541692 PMC4991899

[B191] Sheng Z. Xu J. Wang M. Xu X. Zhu J. Zeng S. . (2025). The role of urinary microbiota in primary and recurrent bladder cancer: insights from a propensity score matching study. BMC Cancer. 25, 468. doi: 10.1186/s12885-025-13817-6, PMID: 40087655 PMC11907829

[B192] Shimada T. Fujii-Kuriyama Y. (2004). Metabolic activation of polycyclic aromatic hydrocarbons to carcinogens by cytochromes P450 1A1 and 1B1. Cancer Sci. 95, 1–6. doi: 10.1111/j.1349-7006.2004.tb03162.x, PMID: 14720319 PMC11158916

[B193] Sloan F. A. Yashkin A. P. Akushevich I. Inman B. A. (2020). The cost to medicare of bladder cancer care. Eur. Urol Oncol. 3, 515–522. doi: 10.1016/j.euo.2019.01.015, PMID: 31412015

[B194] Stading R. Chu C. Couroucli X. Lingappan K. Moorthy B. (2020). Molecular role of cytochrome P4501A enzymes inoxidative stress. Curr. Opin. Toxicol. 20-21, 77–84. doi: 10.1016/j.cotox.2020.07.001, PMID: 33283080 PMC7709944

[B195] Stading R. Gastelum G. Chu C. Jiang W. Moorthy B. (2021). Molecular mechanisms of pulmonary carcinogenesis by polycyclic aromatic hydrocarbons (PAHs): Implications for human lung cancer. Semin. Cancer Biol. 76, 3–16. doi: 10.1016/j.semcancer.2021.07.001, PMID: 34242741 PMC8728691

[B196] Sun S. C. (2017). The non-canonical NF-κB pathway in immunity and inflammation. Nat. Rev. Immunol. 17, 545–558. doi: 10.1038/nri.2017.52, PMID: 28580957 PMC5753586

[B197] Sundi D. Collier K. A. Yang Y. Diaz D. A. Pohar K. S. Singer E. A. . (2024). Roles of androgen receptor signaling in urothelial carcinoma. Cancers (Basel) 16:746. doi: 10.3390/cancers16040746, PMID: 38398136 PMC10886823

[B198] Taniguchi K. Karin M. (2018). NF-κB, inflammation, immunity and cancer: coming of age. Nat. Rev. Immunol. 18, 309–324. doi: 10.1038/nri.2017.142, PMID: 29379212

[B199] Tao L. Day B. W. Hu B. Xiang Y. B. Wang R. Stern M. C. . (2013). Elevated 4-aminobiphenyl and 2,6-dimethylaniline hemoglobin adducts and increased risk of bladder cancer among lifelong nonsmokers–The Shanghai Bladder Cancer Study. Cancer Epidemiol. Biomarkers Prev. 22, 937–945. doi: 10.1158/1055-9965.Epi-12-1447, PMID: 23539508 PMC4065796

[B200] Tchounwou P. B. Centeno J. A. Patlolla A. K. (2004). Arsenic toxicity, mutagenesis, and carcinogenesis–a health risk assessment and management approach. Mol. Cell Biochem. 255, 47–55. doi: 10.1023/b:mcbi.0000007260.32981.b9, PMID: 14971645

[B201] Tessmer I. Margison G. P. (2023). The DNA alkyltransferase family of DNA repair proteins: common mechanisms, diverse functions. Int. J. Mol. Sci. 25:463. doi: 10.3390/ijms25010463, PMID: 38203633 PMC10779285

[B202] Thier R. Brüning T. Roos P. H. Rihs H. P. Golka K. Ko Y. . (2003). Markers of genetic susceptibility in human environmental hygiene and toxicology: the role of selected CYP, NAT and GST genes. Int. J. Hyg Environ. Health 206, 149–171. doi: 10.1078/1438-4639-00209, PMID: 12872524

[B203] Toyooka T. Yanagiba Y. Ibuki Y. Wang R. S. (2018). Trichloroethylene exposure results in the phosphorylation of histone H2AX in a human hepatic cell line through cytochrome P450 2E1-mediated oxidative stress. J. Appl. Toxicol. 38, 1224–1232. doi: 10.1002/jat.3632, PMID: 29722447

[B204] Trédaniel J. Boffetta P. Saracci R. Hirsch A. (1993). Environmental tobacco smoke and the risk of cancer in adults. Eur. J. Cancer 29a, 2058–2068. doi: 10.1016/0959-8049(93)90471-q, PMID: 8280502

[B205] Tsai T. L. Kuo C. C. Hsu L. I. Tsai S. F. Chiou H. Y. Chen C. J. . (2021). Association between arsenic exposure, DNA damage, and urological cancers incidence: A long-term follow-up study of residents in an arseniasis endemic area of northeastern Taiwan. Chemosphere. 266, 129094. doi: 10.1016/j.chemosphere.2020.129094, PMID: 33310355

[B206] Tsai M. L. Tsai Y. G. Lin Y. C. Hsu Y. L. Chen Y. T. Tsai M. K. . (2021). IL-25 induced ROS-mediated M2 macrophage polarization via AMPK-associated mitophagy. Int. J. Mol. Sci. 23. doi: 10.3390/ijms23010003, PMID: 35008429 PMC8744791

[B207] Tseng C. Y. Wang J. S. Chao M. W. (2017). Causation by diesel exhaust particles of endothelial dysfunctions in cytotoxicity, pro-inflammation, permeability, and apoptosis induced by ROS generation. Cardiovasc. Toxicol. 17, 384–392. doi: 10.1007/s12012-016-9364-0, PMID: 26965709

[B208] Tsuji J. S. Alexander D. D. Perez V. Mink P. J. (2014). Arsenic exposure and bladder cancer: quantitative assessment of studies in human populations to detect risks at low doses. Toxicology. 317, 17–30. doi: 10.1016/j.tox.2014.01.004, PMID: 24462659

[B209] Uno Y. Uehara S. Yamazaki H. (2022). Drug-oxidizing and conjugating non-cytochrome P450 (non-P450) enzymes in cynomolgus monkeys and common marmosets as preclinical models for humans. Biochem. Pharmacol. 197, 114887. doi: 10.1016/j.bcp.2021.114887, PMID: 34968483

[B210] Van de Wiele T. Vanhaecke L. Boeckaert C. Peru K. Headley J. Verstraete W. . (2005). Human colon microbiota transform polycyclic aromatic hydrocarbons to estrogenic metabolites. Environ. Health Perspect. 113, 6–10. doi: 10.1289/ehp.7259, PMID: 15626640 PMC1253702

[B211] Verma N. Pink M. Boland S. Rettenmeier A. W. Schmitz-Spanke S. (2017). Benzo[a]pyrene-induced metabolic shift from glycolysis to pentose phosphate pathway in the human bladder cancer cell line RT4. Sci. Rep. 7, 9773. doi: 10.1038/s41598-017-09936-1, PMID: 28851999 PMC5575001

[B212] Vikram H. P. R. Kumar T. P. Kumar G. Beeraka N. M. Deka R. Suhail S. M. . (2024). Nitrosamines crisis in pharmaceuticals - Insights on toxicological implications, root causes and risk assessment: A systematic review. J. Pharm. Anal. 14, 100919. doi: 10.1016/j.jpha.2023.12.009, PMID: 38799236 PMC11126534

[B213] Vinceti M. Filippini T. Del Giovane C. Dennert G. Zwahlen M. Brinkman M. . (2018). Selenium for preventing cancer. Cochrane Database Syst. Rev. 1, Cd005195. doi: 10.1002/14651858.CD005195.pub4, PMID: 29376219 PMC6491296

[B214] Vineis P. Pirastu R. (1997). Aromatic amines and cancer. Cancer Causes Control. 8, 346–355. doi: 10.1023/a:1018453104303, PMID: 9498898

[B215] Vlaanderen J. Straif K. Ruder A. Blair A. Hansen J. Lynge E. . (2014). Tetrachloroethylene exposure and bladder cancer risk: a meta-analysis of dry-cleaning-worker studies. Environ. Health Perspect. 122, 661–666. doi: 10.1289/ehp.1307055, PMID: 24659585 PMC4080536

[B216] Wang S. Hanna D. Sugamori K. S. Grant D. M. (2019). Primary aromatic amines and cancer: Novel mechanistic insights using 4-aminobiphenyl as a model carcinogen. Pharmacol. Ther. 200, 179–189. doi: 10.1016/j.pharmthera.2019.05.004, PMID: 31075357

[B217] Wang H. Liu B. Chen H. Xu P. Xue H. Yuan J. (2023). Dynamic changes of DNA methylation induced by benzo(a)pyrene in cancer. Genes Environ. 45, 21. doi: 10.1186/s41021-023-00278-1, PMID: 37391844 PMC10314634

[B218] Wang B. Qiu Y. Xie M. Huang P. Yu Y. Sun Q. . (2024). Gut microbiota Parabacteroides distasonis enchances the efficacy of immunotherapy for bladder cancer by activating anti-tumor immune responses. BMC Microbiol. 24, 237. doi: 10.1186/s12866-024-03372-8, PMID: 38961326 PMC11221038

[B219] Wang Z. Yang P. Xie J. Lin H. P. Kumagai K. Harkema J. . (2020). Arsenic and benzo[a]pyrene co-exposure acts synergistically in inducing cancer stem cell-like property and tumorigenesis by epigenetically down-regulating SOCS3 expression. Environ. Int. 137, 105560. doi: 10.1016/j.envint.2020.105560, PMID: 32062438 PMC7099608

[B220] Ward M. H. Sinha R. Heineman E. F. Rothman N. Markin R. Weisenburger D. D. . (1997). Risk of adenocarcinoma of the stomach and esophagus with meat cooking method and doneness preference. Int. J. Cancer. 71, 14–19. doi: 10.1002/(sici)1097-0215(19970328)71:1<14::aid-ijc4>3.0.co;2-6, PMID: 9096659

[B221] Wéber A. Vignat J. Shah R. Morgan E. Laversanne M. Nagy P. . (2024). Global burden of bladder cancer mortality in 2020 and 2040 according to GLOBOCAN estimates. World J. Urol. 42, 237. doi: 10.1007/s00345-024-04949-8, PMID: 38625417 PMC11021283

[B222] Weiss N. S. (1995). Cancer in relation to occupational exposure to perchloroethylene. Cancer Causes Control. 6, 257–266. doi: 10.1007/bf00051797, PMID: 7612805

[B223] Weltman M. D. Farrell G. C. Liddle C. (1996). Increased hepatocyte CYP2E1 expression in a rat nutritional model of hepatic steatosis with inflammation. Gastroenterology. 111, 1645–1653. doi: 10.1016/s0016-5085(96)70028-8, PMID: 8942745

[B224] Wiench K. Frei E. Schroth P. Wiessler M. (1992). 1-C-glucuronidation of N-nitrosodiethylamine and N-nitrosomethyl-n-pentylamine *in vivo* and in primary hepatocytes from rats pretreated with inducers. Carcinogenesis. 13, 867–872. doi: 10.1093/carcin/13.5.867, PMID: 1587001

[B225] Wigner P. Grębowski R. Bijak M. Saluk-Bijak J. Szemraj J. (2021). The interplay between oxidative stress, inflammation and angiogenesis in bladder cancer development. Int. J. Mol. Sci. 22:4483. doi: 10.3390/ijms22094483, PMID: 33923108 PMC8123426

[B226] Williams J. R. Jr. Grantham P. H. Marsh H. H. 3rd Weisburger J. H. Weisburger E. K. (1970). Participation of liver fractions and of intestinal bacteria in the metabolism of N-hydroxy-N-2-fluorenylacetamide in the rat. Biochem. Pharmacol. 19, 173–188. doi: 10.1016/0006-2952(70)90338-2, PMID: 4927412

[B227] Wong J. Y. Y. Fischer A. H. Baris D. Beane Freeman L. E. Karagas M. R. Schwenn M. . (2024). Urinary mutagenicity and bladder cancer risk in northern New England. Environ. Mol. Mutagen 65, 47–54. doi: 10.1002/em.22588, PMID: 38465801 PMC11089907

[B228] Wu K. L. Berger T. (2007). Trichloroethylene metabolism in the rat ovary reduces oocyte fertilizability. Chem. Biol. Interact. 170, 20–30. doi: 10.1016/j.cbi.2007.06.038, PMID: 17673192 PMC2085368

[B229] Wu H. Zhao X. Hochrein S. M. Eckstein M. Gubert G. F. Knöpper K. . (2023). Mitochondrial dysfunction promotes the transition of precursor to terminally exhausted T cells through HIF-1α-mediated glycolytic reprogramming. Nat. Commun. 14, 6858. doi: 10.1038/s41467-023-42634-3, PMID: 37891230 PMC10611730

[B230] Wynder E. L. Goldsmith R. (1977). The epidemiology of bladder cancer: a second look. Cancer. 40, 1246–1268. doi: 10.1002/1097-0142(197709)40:3<1246::aid-cncr2820400340>3.0.co;2-5 332323

[B231] Xu S. Y. Chen Y. X. Wu W. X. Wang K. X. Lin Q. Liang X. Q. (2006). Enhanced dissipation of phenanthrene and pyrene in spiked soils by combined plants cultivation. Sci. Total Environ. 363, 206–215. doi: 10.1016/j.scitotenv.2005.05.030, PMID: 15985280

[B232] Xu H. Heinze T. M. Paine D. D. Cerniglia C. E. Chen H. (2010). Sudan azo dyes and Para Red degradation by prevalent bacteria of the human gastrointestinal tract. Anaerobe. 16, 114–119. doi: 10.1016/j.anaerobe.2009.06.007, PMID: 19580882 PMC5863247

[B233] Yamamoto T. Gi M. Yamashita S. Suzuki S. Fujioka M. Vachiraarunwong A. . (2023). DNA methylation aberrations in dimethylarsinic acid-induced bladder carcinogenesis. Cancers (Basel) 15:5274. doi: 10.3390/cancers15215274, PMID: 37958445 PMC10648661

[B234] Yamamoto A. Kawashima A. Uemura T. Nakano K. Matsushita M. Ishizuya Y. . (2025). A novel mouse model of upper tract urothelial carcinoma highlights the impact of dietary intervention on gut microbiota and carcinogenesis prevention despite carcinogen exposure. Int. J. Cancer. 156, 1439–1456. doi: 10.1002/ijc.35295, PMID: 39693209 PMC11789449

[B235] Yan L. Anderson G. M. DeWitte M. Nakada M. T. (2006). Therapeutic potential of cytokine and chemokine antagonists in cancer therapy. Eur. J. Cancer. 42, 793–802. doi: 10.1016/j.ejca.2006.01.013, PMID: 16524718

[B236] Yang H. Jin C. Li J. Zhang Z. Zhao K. Yin X. . (2024). Causal relationship between bladder cancer and gut microbiota contributes to the gut-bladder axis: A two-sample Mendelian randomization study. Urol Oncol. 43:e9–267.e18. doi: 10.1016/j.urolonc.2024.10.014, PMID: 39489648

[B237] Yang M. Mao H. Li H. Yang F. Cao F. Yan W. (2023). Quantifying concentrations and emissions of hexachlorobutadiene - A new atmospheric persistent organic pollutant in northern China. Environ. Res. 216, 114139. doi: 10.1016/j.envres.2022.114139, PMID: 36084678

[B238] Yu Y. Li X. Liang C. Tang J. Qin Z. Wang C. . (2016). The relationship between GSTA1, GSTM1, GSTP1, and GSTT1 genetic polymorphisms and bladder cancer susceptibility: A meta-analysis. Med. (Baltimore). 95, e4900. doi: 10.1097/md.0000000000004900, PMID: 27631264 PMC5402607

[B239] Yu E. Y. W. Wesselius A. Mehrkanoon S. Brinkman M. van den Brandt P. White E. . (2020). Grain and dietary fiber intake and bladder cancer risk: a pooled analysis of prospective cohort studies. Am. J. Clin. Nutr. 112, 1252–1266. doi: 10.1093/ajcn/nqaa215, PMID: 32778880 PMC7657329

[B240] Zappavigna S. Cossu A. M. Grimaldi A. Bocchetti M. Ferraro G. A. Nicoletti G. F. . (2020). Anti-inflammatory drugs as anticancer agents. Int. J. Mol. Sci. 21:2605. doi: 10.3390/ijms21072605, PMID: 32283655 PMC7177823

[B241] Zarerad E. Niksalehi K. Armandeh M. Sani M. A. Ataei M. Mousavi T. . (2023). Polychlorinated biphenyls: A review of recent updates on food safety and environmental monitoring, health and toxicological implications, and analysis. Mini Rev. Med. Chem. 23, 1390–1411. doi: 10.2174/1389557523666221213091445, PMID: 36515022

[B242] Zhang Y. Liu J. (2011). Transgenic alfalfa plants co-expressing glutathione S-transferase (GST) and human CYP2E1 show enhanced resistance to mixed contaminates of heavy metals and organic pollutants. J. Hazard Mater 189, 357–362. doi: 10.1016/j.jhazmat.2011.02.042, PMID: 21411224

[B243] Zhang Y. Liu J. Zhou Y. Gong T. Wang J. Ge Y. (2013). Enhanced phytoremediation of mixed heavy metal (mercury)-organic pollutants (trichloroethylene) with transgenic alfalfa co-expressing glutathione S-transferase and human P450 2E1. J. Hazard Mater. 260, 1100–1107. doi: 10.1016/j.jhazmat.2013.06.065, PMID: 23933506

[B244] Zhang S. Ma J. Ma Y. Yi J. Wang B. Wang H. . (2024). Engineering probiotics for diabetes management: advances, challenges, and future directions in translational microbiology. Int. J. Nanomedicine. 19, 10917–10940. doi: 10.2147/ijn.S492651, PMID: 39493275 PMC11530765

[B245] Zhang Z. F. Sarkis A. S. Cordon-Cardo C. Dalbagni G. Melamed J. Aprikian A. . (1994). Tobacco smoking, occupation, and p53 nuclear overexpression in early stage bladder cancer. Cancer Epidemiol. Biomarkers Prev. 3, 19–24., PMID: 8118380

[B246] Zhang H. Shen Y. Liu W. He Z. Fu J. Cai Z. . (2019). A review of sources, environmental occurrences and human exposure risks of hexachlorobutadiene and its association with some other chlorinated organics. Environ. pollut. 253, 831–840. doi: 10.1016/j.envpol.2019.07.090, PMID: 31344544

[B247] Zhang Y. Talalay P. (1994). Anticarcinogenic activities of organic isothiocyanates: chemistry and mechanisms. Cancer Res. 54, 1976s–1981s. 8137323

[B248] Zhao C. Jin H. Lei Y. Li Q. Zhang Y. Lu Q. (2024a). The dual effects of Benzo(a)pyrene/Benzo(a)pyrene-7,8-dihydrodiol-9,10-epoxide on DNA Methylation. Sci. Total Environ. 950, 175042. doi: 10.1016/j.scitotenv.2024.175042, PMID: 39084379

[B249] Zhao H. Lin J. Grossman H. B. Hernandez L. M. Dinney C. P. Wu X. (2007). Dietary isothiocyanates, GSTM1, GSTT1, NAT2 polymorphisms and bladder cancer risk. Int. J. Cancer. 120, 2208–2213. doi: 10.1002/ijc.22549, PMID: 17290402

[B250] Zhao C. Yang L. Sun Y. Chen C. Huang Z. Yang Q. . (2024b). Atmospheric emissions of hexachlorobutadiene in fine particulate matter from industrial sources. Nat. Commun. 15, 4737. doi: 10.1038/s41467-024-49097-0, PMID: 38834556 PMC11150375

[B251] Zheng R. Gao F. Mao Z. Xiao Y. Yuan L. Huang Z. . (2023). LncRNA BCCE4 genetically enhances the PD-L1/PD-1 interaction in smoking-related bladder cancer by modulating miR-328-3p-USP18 signaling. Adv. Sci. (Weinh). 10, e2303473. doi: 10.1002/advs.202303473, PMID: 37705121 PMC10602555

[B252] Zhu L. Jia X. Xie H. Zhang J. Zhu Q. (2024). Trichloroethylene exposure, multi-organ injury, and potential mechanisms: A narrative review. Sci. Total Environ. 946, 174029. doi: 10.1016/j.scitotenv.2024.174029, PMID: 38944297

[B253] Zou R. Lu J. Bai X. Yang Y. Zhang S. Wu S. . (2024). The epigenetic-modified downregulation of LOXL1 protein mediates EMT in bladder epithelial cells exposed to benzo[a]pyrene and its metabolite BPDE. Int. Immunopharmacol 142, 113232. doi: 10.1016/j.intimp.2024.113232, PMID: 39340995

