# Supplementary material for: Carcinogen metabolism and bladder cancer: role of gut microbiota in disease and prevention
Source: Front Cell Infect Microbiol. 2026 Jan 30;15:1727550. doi: 10.3389/fcimb.2025.1727550 (PMC12901428; doi:10.3389/fcimb.2025.1727550)
Supplement: Supplementary file 1 [file Table1.docx]

Supplementary Table 1: Carcinogens metabolized by the GI microbiota

| Carcinogenic compounds | | | Enzyme sources | Results | Reference |
| --- | --- | --- | --- | --- | --- |
| PAHs | Benzo(a)-pyrene | Found in diesel engine emission, cooked meat | SHIME inoculated with human feces | Colonic digests became estrogenic. 7-OH benzo(a)pyrene detected in colonic digests. | (Van de Wiele et al. 2005) [1] |
|  | Naphthalene | Insecticide, used as precursor for plasticizer synthesis | SHIME inoculated with human feces | Colonic digests became estrogenic. | (Van de Wiele et al. 2005) [1] |
|  |  |  | Bile cannulated rats, GF vs. conv rats | Enterohepatic circulation of naphtalene glutathion conjugates. Microbiota involved in the production of CH_3_S-metabolites and the naphthols. | (Bakke et al. 1985) [2] |
|  | Phenanthrene | Found in cigarette smoke | SHIME inoculated with human feces | Colonic digests became estrogenic. | (Van de Wiele et al. 2005) [1] |
|  | Pyrene | Used as dye and dye precursor | SHIME inoculated with human feces | Colonic digests became estrogenic. 1-OH pyrene detected in colonic digests. | (Van de Wiele et al. 2005) [1] |
| Nitro-PAHs | 2-nitrofluorene | By-product of combustion | GF and conv rats treated orally | 7-, 5-, 3-, 1-OH acetylaminofluorene major metabolites in conv rats, while only OH-NF in GF rats. Mutagenicity increased in urine and feces of GF rats. | (Möller et al. 1988) [3] |
|  |  |  | GF and conv rats treated orally | More DNA adducts in conv animals in all tissues studied. | (Möller et al. 1994) [4] |
|  | 6-nitrobenzo(a)- pyrene | Found in engine emission | Fecal suspensions (human and rat) | Reduced to 6-aminobenzo(a)pyrene | (Cerniglia et al. 1984) [5] |
|  |  |  | Human fecal suspensions (anaerobic) | Reduced to 6-nitrosobenzo(a)pyrene and 6-aminobenzo(a)pyrene. 6-nitrosobenzo[a]pyrene exhibited strong direct-acting mutagenicity. | (Fu et al. 1988) [6] |
|  | 3-nitrobenzo(a)-pyrene | Found in engine emission | Rat fecal suspensions (anaerobic) | Reduced as 3-aminobenzo(a)pyrene | (Richardson et al. 1988) [7] |
|  | 6-nitrochrysene | Found in ambient air | SHIME inoculated with human feces. Fecal suspensions (human, mouse, rat) | Reduced to 6-aminochrysene | (Manning et al. 1988) [8] |
| Metals | Arsenic (As) | Ubiquitous environmental contaminant | Feacal suspensions (human); SHIME | Both inorganic As and soil-contaminated As were metabolized into methylated arsenicals and thioarsenicals | (Van de Wiele et al. 2010) [9] |
|  |  |  | WT vs. MDR2-deficient rats | Generation of thioarsenicals dependant of enterohepatic ciculation. Arsenicals excreted from bile transformed into MMMTA(V) and DMMTA(V) | (Bu et al. 2011) [10] |
| PCBs | 2,4',5- trichlorobiphenyl | Were widely used as dielectric and coolant fluids | Bile cannulated rats, GF v. conv rats | Fat from conv rats contained 3-15 times more radioactivity than GF. 95% of radioactivity detected in bile of conv rats. | (Gustafsson et al. 1981) [11] |
|  |  |  | GF vs. conv mice | 4-MeSO2-triCB higher in lung, kidneys and liver of conv mice | (Brandt et al. 1982) [12] |
| Pesticides | DDT | Organochlorine pesticide | Eubacterium limosum isolated from human GI microbiota | Metabolized to 1,1-dichloro-2,2-bis(pchlorophenyl)ethane (DDD) | (Yim et al. 2008) [13] |
|  |  |  | Rats injected by stomach tube or ip; cultures of E. Coli or A. aerogenes isolated from rat feces | DDD detected in rats receiving DDT by stomach tube not ny ip. DDD detected in bacterial cultures. | (Mendel and Walton 1966) [14] |
|  | Lindane | Organochlorine pesticide | Cecal content (rat) | Dechlorinated to 3,4,5,6-Tetrachlorocyclohex-1-ene | (Stein et al. 1980) [15] |
| Azo dyes | Azobenzene | Colorants in food products | GF vs conv rats | Reduced to aniline only in conv. rats. | (Macholz et al. 1985) [16] |
|  | Sudan I, II, III, IV and ParaRed |  | Human fecal suspensions (anaerobic) | Metabolized to aniline, 2,4-dimethylaniline, o-toluidine, and 4-nitroaniline. | (Xu et al. 2007) [17] |
|  | Sudan I, II, III, IV |  | Human intestinal bacteria (isolated strains) | Bifidobacterium infantis, Clostridium indolis, Enterococcus faecalis, Lactobacillus rhamnosus and Ruminococcus obeum able to reduce the 4 dyes. Escherichia coli and Peptostreptococcus magnus not able to reduce any of the 4 dyes. | (Xu et al. 2010) [18] |

PAHs, polycyclic aromatic hydrocarbons; PCBs, polychlorobiphenyls; SHIME, Simulator of the Human Intestinal Microbial Ecosystem.

[1] T. Van de Wiele, L. Vanhaecke, C. Boeckaert, K. Peru, J. Headley, W. Verstraete, S. Siciliano, *Environ Health Perspect* **2005**, *113* (1), 6, <https://doi.org/10.1289/ehp.7259>.

[2] J. Bakke, C. Struble, J. A. Gustafsson, B. Gustafsson, *Proc Natl Acad Sci U S A* **1985**, *82* (3), 668, <https://doi.org/10.1073/pnas.82.3.668>.

[3] L. Möller, M. Corrie, T. Midtvedt, J. Rafter, J. A. Gustafsson, *Carcinogenesis* **1988**, *9* (5), 823, <https://doi.org/10.1093/carcin/9.5.823>.

[4] L. Möller, M. Zeisig, T. Midtvedt, J. A. Gustafsson, *Carcinogenesis* **1994**, *15* (5), 857, <https://doi.org/10.1093/carcin/15.5.857>.

[5] C. E. Cerniglia, P. C. Howard, P. P. Fu, W. Franklin, *Biochem Biophys Res Commun* **1984**, *123* (1), 262, <https://doi.org/10.1016/0006-291x(84)90407-8>.

[6] P. P. Fu, C. E. Cerniglia, K. E. Richardson, R. H. Heflich, *Mutat Res* **1988**, *209* (3-4), 123, <https://doi.org/10.1016/0165-7992(88)90028-0>.

[7] K. E. Richardson, P. P. Fu, C. E. Cerniglia, *J Toxicol Environ Health* **1988**, *23* (4), 527, <https://doi.org/10.1080/15287398809531134>.

[8] B. W. Manning, W. L. Campbell, W. Franklin, K. B. Delclos, C. E. Cerniglia, *Appl Environ Microbiol* **1988**, *54* (1), 197, <https://doi.org/10.1128/aem.54.1.197-203.1988>.

[9] T. Van de Wiele, C. M. Gallawa, K. M. Kubachka, J. T. Creed, N. Basta, E. A. Dayton, S. Whitacre, G. Du Laing, K. Bradham, *Environ Health Perspect* **2010**, *118* (7), 1004, <https://doi.org/10.1289/ehp.0901794>.

[10] N. Bu, H. Y. Wang, W. H. Hao, X. Liu, S. Xu, B. Wu, Y. Anan, Y. Ogra, Y. J. Lou, H. Naranmandura, *Metallomics* **2011**, *3* (10), 1064, <https://doi.org/10.1039/c1mt00036e>.

[11] J. A. Gustafsson, J. J. Rafter, J. E. Bakke, B. E. Gustafsson, *Nutr Cancer* **1981**, *2* (4), 224, <https://doi.org/10.1080/01635588109513687>.

[12] I. Brandt, E. Klasson-Wehler, J. Rafter, A. Bergman, *Toxicol Lett* **1982**, *12* (4), 273, <https://doi.org/10.1016/0378-4274(82)90251-x>.

[13] Y. J. Yim, J. Seo, S. I. Kang, J. H. Ahn, H. G. Hur, *Arch Environ Contam Toxicol* **2008**, *54* (3), 406, <https://doi.org/10.1007/s00244-007-9044-y>.

[14] J. L. Mendel, M. S. Walton, *Science* **1966**, *151* (3717), 1527, <https://doi.org/10.1126/science.151.3717.1527>.

[15] K. Stein, J. Portig, H. Fuhrmann, W. Koransky, G. Noack, *Xenobiotica* **1980**, *10* (1), 65, <https://doi.org/10.3109/00498258009033732>.

[16] R. Macholz, M. Kujawa, J. Schulze, H. J. Lewerenz, W. Schnaak, *Arch Toxicol Suppl* **1985**, *8*, 373, <https://doi.org/10.1007/978-3-642-69928-3_77>.

[17] H. Xu, T. M. Heinze, S. Chen, C. E. Cerniglia, H. Chen, *Appl Environ Microbiol* **2007**, *73* (23), 7759, <https://doi.org/10.1128/aem.01410-07>.

[18] H. Xu, T. M. Heinze, D. D. Paine, C. E. Cerniglia, H. Chen, *Anaerobe* **2010**, *16* (2), 114, <https://doi.org/10.1016/j.anaerobe.2009.06.007>.
